# Heat-shock protein 90α is a potential prognostic and predictive biomarker in hepatocellular carcinoma: a large-scale and multicenter study

**DOI:** 10.1007/s12072-022-10391-y

**Published:** 2022-08-16

**Authors:** Ke Su, Yanlin Liu, Pan Wang, Kun He, Fei Wang, Hao Chi, Mingyue Rao, Xueting Li, Lianbin Wen, Yanqiong Song, Jianwen Zhang, Tao Gu, Ke Xu, Qi Li, Jiali Chen, Zhenying Wu, Han Li, Weihong Huang, Lan Chen, Jian Tong, Hongyan Li, Xunjie Feng, Siyu Chen, Binbin Yang, Hongping Jin, Yue Yang, Hanlin Liu, Chao Yang, Ming Wu, Fangyu Xiong, Keyi Peng, Lechuan Zhu, Yaoyang Xu, Xue Tang, Zunyuan Tan, Xiaotong Luo, Hanyue Zheng, Yuxin Zhang, Lu Guo, Yunwei Han

**Affiliations:** 1 Department of Oncology, The Affiliated Hospital of Southwest Medical University, 25 Taiping Street, Luzhou, 46000 Sichuan China; 2 Clinical Skills Center, The Affiliated Hospital of Southwest Medical University, Luzhou, 646000 China; 3 Clinical Research Institute, The Affiliated Hospital of Southwest Medical University, Luzhou, 646000 China; 4 Department of General Surgery, Luxian People’s Hospital, Luzhou, 646199 China; 5 Clinical Medical College, Southwest Medical University, Luzhou, 646000 China; 6 Department of Oncology, 363 Hospital, Chengdu, 610041 China; 7 Department of Geriatric Cardiology, Sichuan Academy of Medical Sciences and Sichuan Provincial People’s Hospital, Chengdu, 610072 China; 8 Department of Radiotherapy, Sichuan Cancer Hospital and Institute, Sichuan Cancer Center, School of Medicine, University of Electronic Science and Technology of China, Chengdu, 610042 China; 9 Department of Oncology and Hematology, The Affiliated Traditional Chinese Medicine Hospital of Southwest Medical University, Luzhou, 646000 China; 10 Department of Spinal Surgery, No.1 Orthopedics Hospital of Chengdu, Chengdu, 610000 China; 11 Department of Anesthesiology, Affiliated Traditional Chinese Medicine Hospital of Southwest Medical University, Luzhou, 646000 China; 12 Department of Medical Inspection Technology, Southwest Medical University, Luzhou, 646000 China; 13 Department of Ophthalmology, The Affiliated Hospital of Southwest Medical University, 25 Taiping Street, Luzhou, 646000 Sichuan China; 14 Nuclear Medicine and Molecular Imaging Key Laboratory of Sichuan Province, Luzhou, 646000 China; 15 Academician (Expert) Workstation of Sichuan Province, Luzhou, 646000 China

**Keywords:** Heat-shock protein 90α, Hepatocellular carcinoma, Biomarker, Overall survival, Primary liver cancer, Transarterial chemoembolization, Immune checkpoint inhibitor, Targeted therapy, Prognostic factor, Predictive biomarker

## Abstract

**Background:**

Although the diagnostic value of plasma heat-shock protein 90α (HSP90α) in hepatocellular carcinoma (HCC) has been previously reported, the causal effect of the plasma HSP90α levels on HCC prognosis remains largely unclear. To this extent, we sought to assess whether the plasma HSP90α acts as a prognostic factor for HCC patients.

**Methods:**

A total of 2150 HCC patients were included in this retrospective study between August 2016 and July 2021. Plasma HSP90α levels were tested within a week before treatment and their association with prognosis was assessed.

**Results:**

An optimal cutoff value of 143.5 for the HSP90α based on the overall survival (OS) was determined using the X-tile software. HCC patients with HSP90α < 143.5 ng/mL (low HSP90α) before and after propensity score matching (PSM) indicated longer median OS (mOS) relative to those with HSP90α ≥ 143.5 ng/mL (high HSP90α) (37.0 vs. 9.0 months, *p* < 0.001; 19.2 vs. 9.6 months, *p* < 0.001; respectively). In addition, the high HSP90α plasma level is an independent poor prognostic factor for OS in HCC patients. In our subgroup analysis, including the supportive care group, surgery group, transarterial chemoembolization (TACE) group, adjuvant TACE group, an immune checkpoint inhibitor (ICI) plus targeted therapy group, and TACE plus ICI group, the high HSP90α group demonstrated better OS compared to the low HSP90α group. Moreover, in the supportive care, TACE, ICI plus targeted therapy, TACE plus ICI groups, and high HSP90α levels were also an independent poor prognostic factors for OS.

**Conclusions:**

Our study confirmed that the plasma HSP90α level can be used as a prognostic biomarker for HCC.

**Supplementary Information:**

The online version contains supplementary material available at 10.1007/s12072-022-10391-y.

## Introduction

Hepatocellular carcinoma (HCC) is one of the most common malignant tumors worldwide and a common cause of cancer death [1]. The median overall survival (mOS) of HCC patients without effective treatment is only 4 months [2]. Nowadays, a plethora of therapeutic approaches has been investigated in HCC. Recently, the combination of the anti-program death ligand 1(PD-L1) antibody, atezolizumab plus the anti-VEGF bevacizumab was approved by the Food and Drug Administration (FDA) and recommended by the National Comprehensive Cancer Network (NCCN) guidelines as the first-line treatment for advanced HCC, which could extend OS to 19.2 months [3, 4]. Yusheng et al. reported that the median progression-free survival (mPFS) of advanced HCC patients receiving transarterial chemoembolization (TACE) plus camrelizumab was 9 months [5]. In addition, in a randomized controlled study of operable HCC, patients who received adjuvant TACE had a higher three-year OS rate compared to patients who underwent surgery alone (85.2% vs. 77.4%; *p* = 0.04) [6]. Although the survival of HCC has been greatly prolonged, predicting treatment efficacy and response remains a challenging bottleneck.

In clinical practice, alpha-fetoprotein (AFP) is the most commonly used diagnostic and prognostic marker for HCC [7]. However, its reduced sensitivity of 52.1–62.5% underlies numerous limitations [8, 9]. Furthermore, AFP-negative tumors account for up to 30–40% of pathologically diagnosed HCC patients, which significantly hinders the application of AFP in the diagnosis and prognosis of HCC [10–12]. Therefore, there is an urgent need to identify new prognostic and predictive biomarkers to improve the management of HCC patients.

Heat-shock protein 90 (HSP90) is a highly conserved molecular chaperone through species and evolution. Interestingly, HSP90 has been reported to be secreted by a variety of cancer cell types [13, 14]. Previous studies had demonstrated that the HSP90 expression was associated with tumor proliferation and metastasis [15–17]. HSP90α is a subtype of HSP90, which has become a remarkable focus of current research due to its role in the regulation of signal transduction [18]. In a large multicenter study with 1,647 enrollments for the diagnosis of HCC, HSP90α displayed 92.7% and 91.3% diagnostic sensitivity and specificity, respectively [19]. Nevertheless, despite these promising results, there is still a shortfall of clinical studies, with large sample sizes, to determine the relationship between HSP90α level and HCC prognosis. Therefore, we initiated this multicenter study to assess whether plasma HSP90α could be used as a prognostic factor in HCC patients.

## Materials and methods

### Patients

A total of 2150 HCC patients were initially enrolled at three Chinese tertiary hospitals between August 2016 and July 2021. The inclusion criteria were as follows: (a) pathologically or clinically diagnosed HCC; (b) no prior anti-tumor therapy; (c) presence of measurable lesions according to Response Evaluation Criteria in Solid Tumors, version 1.1 (RECIST 1.1); and (d) plasma HSP90α test completed within a week before treatment. Patients with other malignant tumors or incomplete clinical data were excluded. The Ethics Committee of The Affiliated Hospital of Southwest Medical University approved this study with the affiliated approval number KY2020254. Due to the retrospective nature of the study, informed consent was waived.

### Data collection

We retrospectively reviewed and recorded clinical data through individual patients' files. Demographic information included sex and age. HCC etiology factors of interest included alcohol, hepatitis B virus (HBV), hepatitis C virus (HCV), and nonalcoholic fatty liver disease (NAFLD). The patient’s liver function was evaluated using the Child–Pugh score and albumin–bilirubin (ALBI). Laboratory data included the HSP90α plasma levels, AFP, alkaline phosphatase (ALP), alanine aminotransferase (ALT), lactate dehydrogenase (LDH), total bilirubin, albumin, leukocyte and platelet count, and creatinine. Tumor burden was interpreted by radiologists by computed tomography (CT) and magnetic resonance imaging (MRI), which included the maximum tumor diameter, number of tumors, portal vein tumor thrombus (PVTT), lymph node metastasis, and extrahepatic metastasis. The Barcelona Clinic Liver Cancer (BCLC) staging system was used to determine the tumor stage. All HCC treatments, hypertension, and diabetes were documented based on the patient’s medical record. OS was defined as the time from the start of the first treatment until death or the last follow-up.

### Statistical analysis

For statistical analysis, the Chi-square (*χ*^2^) test and McNemar analysis were used to analyze categorical variables. Mann–Whitney U and Wilcoxon matched-pairs signed-rank tests were used to analyze continuous variables. An optimal cutoff value of the HSP90α levels based on OS was determined using X-tile software (Yale University, New Haven, CT). A time-dependent receiver operating characteristic (ROC) curve was used to assess the ability of HSP90α to predict efficacy. The relationship between the HSP90α level and baseline characteristics was assessed by using a univariate and multivariate logistic regression model. Propensity score matching (PSM) was performed to determine the high and low HSP90α level groups with a similar baseline. Subsequently, the mOS was estimated and compared using Kaplan–Meier statistics and log-rank test, respectively. After identifying Factors affecting OS (*p* < 0.05) via univariate Cox analysis, we introduced them into multivariate models to determine independent prognostic factors for OS. All statistical analyses were carried out in SPSS (version 26.0) and R 3.3.2 software. Two-sided *p* < 0.05 were considered statistically significant.

## Results

### Patient characteristics

A total of 2150 HCC patients were included in our retrospective study. The median HSP90α plasma concentration was 100.4 ng/mL (IQR 56.5–203.5). The percentages of male, child A, AFP < 200 ng/mL, and multiple tumors were 80.3%, 72.8%, 55.1%, and 72.9%, respectively. Most of the patients were presented with BCLC stage C (52.9%) and ALBI grade 2 (60.9%). In addition, the percentages of patients with only supportive care were 21.3%. Table 1 summarizes the baseline characteristics of all enrolled patients.

**Table 1:** Baseline characteristics before propensity score matching

Variable	Total	HSP90α < 143.5 ng/mL	HSP90α ≥ 143.5 ng/mL	*p*
Patients	2150	1370	780	
Male sex	1726 (80.3)	1073 (78.3)	653 (83.7)	0.002
Age ≥ 65 years	569 (26.5)	399 (29.1)	170 (21.8)	< 0.001
Etiology				
HBV	1183 (55.0)	754 (55.0)	429 (55.0)	0.987
HCV	45 (2.1)	31 (2.3)	14 (1.8)	0.466
Alcohol	885 (41.2)	540 (39.4)	345 (44.2)	0.029
NAFLD	30 (1.4)	19 (1.4)	11 (1.4)	0.965
Other	38 (1.8)	24 (1.8)	14 (1.8)	0.942
Diabetes mellitus	206 (9.6)	145 (10.6)	61 (7.8)	0.036
Hypertension	329 (15.3)	227 (16.6)	102 (13.1)	0.031
Child–Pugh class				< 0.001
A	1565 (72.8)	1100 (80.3)	465 (59.6)	
B	553 (25.7)	258 (18.8)	295 (37.8)	
C	32 (1.5)	12 (0.9)	20 (2.6)	
ALBI grade				< 0.001
1	644 (30.0)	508 (37.1)	136 (17.4)	
2	1310 (60.9)	776 (56.6)	534 (68.5)	
3	196 (9.1)	86 (6.3)	110 (14.1)	
HSP90α, median (IQR, ng/mL)	100.4 (56.5–203.5)	66.0 (45.8–95.5)	251.5 (189.9–336.5)	
Creatinine, median (IQR, mg/dL)	64.0 (54.5–73.6)	64.9 (55.0–74.1)	62.2 (53.0–72.3)	0.010
Serum AFP, ng/mL				< 0.001
< 200	1184 (55.1)	887 (64.7)	297 (38.1)	
≥ 200, < 400	139 (6.5)	91 (6.6)	48 (6.2)	
≥ 400	827 (38.5)	392 (28.6)	435 (55.8)	
ALP levels ≥ 125 U/L	1164 (54.1)	568 (41.5)	596 (76.4)	< 0.001
Platelet count ≥ 100 × 109/L	1570 (73.0)	944 (68.9)	626 (80.3)	< 0.001
ALT levels ≥ 40 U/L	1116 (51.9)	613 (44.7)	503 (64.5)	< 0.001
Leukocyte ≥ 4 × 10^9^/L	1791 (83.3)	1087 (79.3)	704 (90.3)	< 0.001
BCLC stage				< 0.001
0/A	486 (22.6)	425 (31.0)	61 (7.8)	
B	494 (23.0)	384 (28.0)	110 (14.1)	
C	1138 (52.9)	549 (40.1)	589 (75.5)	
D	32 (1.5)	12 (0.9)	20 (2.6)	
Number of tumors ≥ 2	1568 (72.9)	920 (67.2)	648 (83.1)	< 0.001
Tumor diameter, cm				< 0.001
< 3	327 (15.2)	286 (20.9)	41 (5.3)	
≥ 3, < 5	467 (21.7)	380 (27.7)	87 (11.2)	
≥ 5, < 10	820 (38.1)	519 (37.9)	301 (38.6)	
≥ 10	536 (24.9)	185 (13.5)	351 (45)	
PVTT	717 (33.3)	284 (20.7)	433 (55.5)	< 0.001
Lymph node metastasis	822 (38.2)	386 (28.2)	436 (55.9)	< 0.001
Extrahepatic metastases	414 (19.3)	196 (14.3)	218 (27.9)	< 0.001
Lung	259 (12.0)	106 (7.7)	153 (19.6)	
Bone	107 (5.0)	58 (4.2)	49 (6.3)	
Other	166 (7.7)	90 (6.6)	76 (9.7)	
Treatments				
Supportive care	457 (21.3)	232 (16.9)	225 (28.8)	< 0.001
Liver resection	489 (22.7)	405 (29.6)	84 (10.8)	< 0.001
Radiotherapy	52 (2.4)	29 (2.1)	23 (2.9)	0.227
TACE	1065 (49.5)	648 (47.3)	417 (53.5)	0.006
RFA	141 (6.6)	128 (9.3)	13 (1.7)	< 0.001
ICI	208 (9.7)	109 (8.0)	99 (12.7)	< 0.001
Targeted therapy	163 (7.6)	92 (6.7)	71 (9.1)	0.044
Chemotherapy	142 (6.6)	108 (7.9)	34 (4.4)	0.002

*HBV* hepatitis B virus, *HCV* hepatitis C virus, *NAFLD* nonalcoholic fatty liver disease, *ALBI* albumin–bilirubin, *HSP90α* heat-shock protein 90α, *AFP* alpha-fetoprotein, *ALP* alkaline phosphatase, *ALT* alanine aminotransferase, *BCLC* Barcelona Clinic Liver Cancer, *PVTT* portal vein tumor thrombus, *TACE* transcatheter arterial chemoembolization, RFA radiofrequency ablation, *ICI* immune checkpoint inhibitor

### HSP90α levels and overall survival before and after PSM

**Fig. 1:**
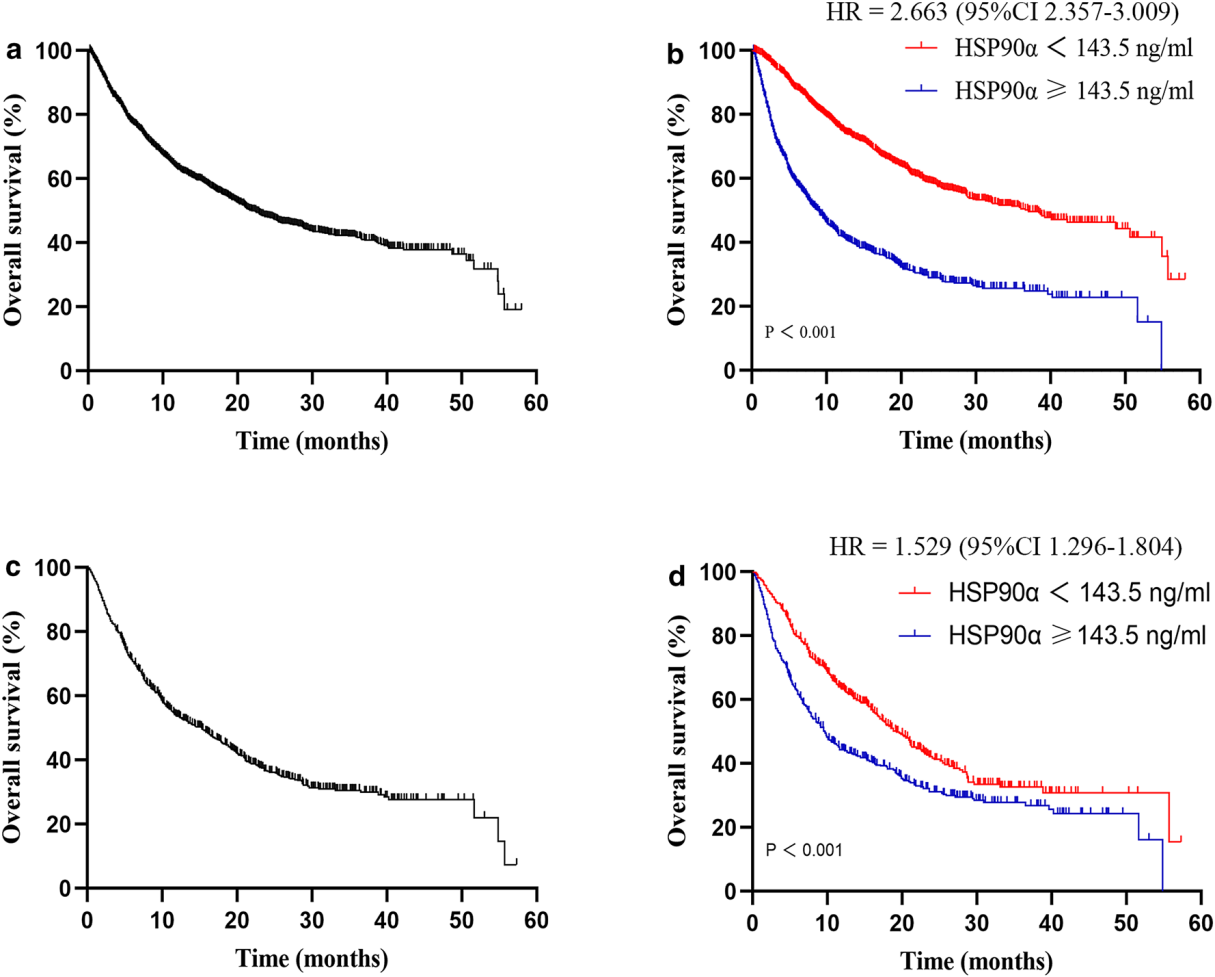
Kaplan–Meier plots: overall survival in all patients **A** stratified based on the HSP90α levels **B** before propensity score matching. Overall survival in matched patients **C** stratified based on the HSP90α levels **D** after propensity score matching. *HSP90α* heat-shock protein 90α

An optimal cutoff value of 143.5 ng/mL for the HSP90α based on OS was determined using X-tile software (Yale University, New Haven, CT). Patients were sub-grouped into high HSP90α (HSP90α ≥ 143.5 ng/mL) and low HSP90α (HSP90α < 143.5 ng/mL) groups. Before PSM, no significant differences were noted between the two groups in terms of the HBV and HCV infection status. However, in the high HSP90α group the patients had older median age, more aggressive baseline BCLC stage, reduced liver function, and elevated tumor burden (*p* < 0.05), compared with the low HSP90α group (Table 1). In this study, the median follow-up was 24.4 months in all patients, 23.7 months in the high HSP90α group, and 24.7 months in the low HSP90α group. The mOS of this HCC patient cohort was 21.9 (95% CI 19.4–24.4) months (Fig. 1A). Patients in the high HSP90α group showed shorter mOS than patients in the low HSP90α group (9.0 vs. 37.0 months, HR = 2.663 (95% CI 2.357–3.009), *p* < 0.001; Fig. 1B).

After PSM, no significant differences were noted between the two groups for any covariate (Supplementary Table 1). The mOS of this HCC patient cohort was 15.3 (95% CI 13.1–17.5) months (Fig. 1C). The high HSP90α group showed a shorter mOS than the low HSP90α group (9.6 vs. 19.2 months, HR = 1.529 (95% CI 1.296–1.804), *p* < 0.001; Fig. 1D).

### Factors associated with the OS

**Fig. 2:**
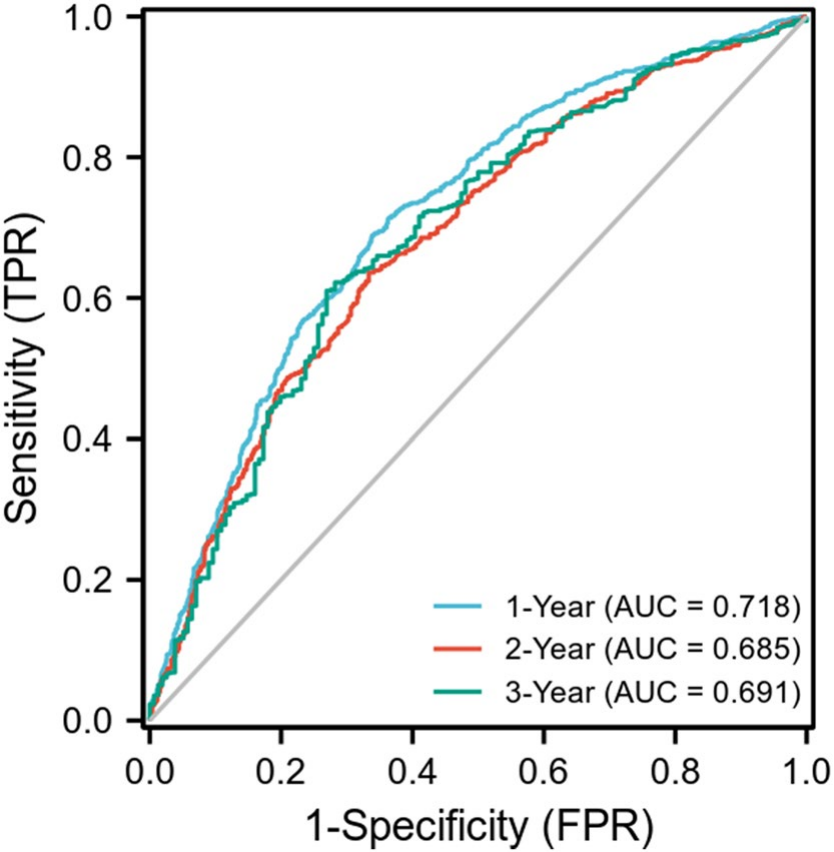
Time-dependent receiver operating characteristic curves of HSP90α for overall survival in hepatocellular carcinoma patients. *HSP90α* heat-shock protein 90α, *AUC* area under the curve

By utilizing univariate and multivariate analyses, we confirmed the HSP90α ≥ 143.5 ng/mL (*p* < 0.001), AFP ≥ 400 ng/mL (*p* = 0.043), child B plus C (*p* = 0.013), ALP ≥ 125 U/L (*p* < 0.001), tumor number ≥ 2 (*p* = 0.010), no any anti-tumor tumors (*p* < 0.001), and more advanced BCLC staging (*p* = 0.006), and these were independent risk prognostic factors for OS (Table 2). After PSM, HSP90α ≥ 143.5 ng/mL remained a negative independent prognostic marker for OS (Supplementary Table 2). In addition, the time-dependent ROC curves based on the HSP90α level demonstrated that the area under the curve (AUC) values for predicting OS at 1, 2, and 3 years was 0.718, 0.685, and 0.691, respectively (Fig. 2).

**Table 2:** Univariate and multivariate Cox regression analysis of overall survival before PSM

	Univariable Cox regression			Multivariable Cox regression		
	HR	95% CI	*p*	HR	95% CI	*p*
Sex (male/female)	1.189	1.015–1.393	0.032	1.113	0.947–1.309	0.194
Age (≥ 65/ < 65 years)	0.979	0.852–1.124	0.763			
HBV (positive/negative)	0.944	0.836–1.066	0.354			
HCV (positive/negative)	0.714	0.442–1.154	0.169			
Alcoholism (positive/negative)	1.092	0.966–1.234	0.160			
NAFLD (positive/negative)	0.859	0.486–1.518	0.602			
Diabetes mellitus (positive/negative)	0.961	0.779–1.186	0.712			
Hypertension (positive/negative)	0.888	0.745–1.060	0.188			
Child–Pugh class (B + C/A)	2.059	1.812–2.340	< 0.001	1.211	1.041–1.409	0.013
ALBI grade (2 + 3/1)	1.594	1.387–1.831	< 0.001	1.069	0.915–1.250	0.400
HSP90α (≥ 143.5/ < 143.5 ng/mL)	2.663	2.357–3.009	< 0.001	1.637	1.418–1.889	< 0.001
AFP (≥ 400/ < 400 ng/mL)	1.520	1.346–1.716	< 0.001	1.142	1.004–1.298	0.043
ALP (≥ 125/ < 125 U/L)	2.345	2.063–2.666	< 0.001	1.431	1.237–1.656	< 0.001
Platelet (< 100,000/ ≥ 100,000/μL)	1.092	0.952–1.252	0.211			
ALT (≥ 40/ < 40 U/L)	1.338	1.184–1.512	< 0.001	0.953	0.836–1.085	0.464
Leukocyte (< 4000/ ≥ 4000/μL)	1.161	0.984–1.370	0.077			
BCLC stage			< 0.001			0.006
0/A	1.000			1.000		
B	1.733	1.388–2.162	< 0.001	1.254	0.950–1.656	0.111
C	3.712	3.070–4.488	< 0.001	1.667	1.239–2.243	0.001
D	6.656	4.332–10.227	< 0.001	1.305	0.796–2.139	0.291
Number of tumor (≥ 2/ < 2)	2.030	1.736–2.374	< 0.001	1.300	1.065–1.586	0.010
Tumor diameter (≥ 5/ < 5 cm)	1.735	1.519–1.981	< 0.001	1.074	0.926–1.247	0.345
PVTT (positive/negative)	2.177	1.924–2.464	< 0.001	1.097	0.929–1.295	0.277
Lymph node metastasis (yes/no)	2.232	1.975–2.523	< 0.001	1.041	0.878–1.234	0.642
Extrahepatic metastases (yes/no)	1.947	1.691–2.241	< 0.001	1.092	0.932–1.278	0.276
Anti-tumor therapy (no/yes)	2.818	2.469–3.216	< 0.001	2.139	1.851–2.472	< 0.001

*PSM* propensity score matching, *HBV* hepatitis B virus, *HCV* hepatitis C virus, *NAFLD* nonalcoholic fatty liver disease, *ALBI* albumin–bilirubin, *HSP90α* heat-shock protein 90α, *AFP* alpha-fetoprotein, *ALP* alkaline phosphatase, *ALT* alanine aminotransferase, *BCLC* Barcelona Clinic Liver Cancer, *PVTT* portal vein tumor thrombus

### Subgroup analysis of different treatment modalities

The patients were divided into different subgroups according to various treatments. The specific subgroups are as follows: supportive care group (*n* = 457), surgery group (*n* = 275), TACE group (*n* = 780), adjuvant TACE group (*n* = 107), immune checkpoint inhibitor (ICI) plus targeted therapy group (*n* = 93), and TACE plus ICI group (*n* = 74). Following this subgrouping, we were willing to elucidate the relationship between HSP90α levels and baseline characteristics in different subgroups, demonstrating that patients with high HSP90α plasma levels were significantly associated with worse tumor burden and more aggressive BCLC staging (Supplementary Tables 3–8).

**Fig. 3:**
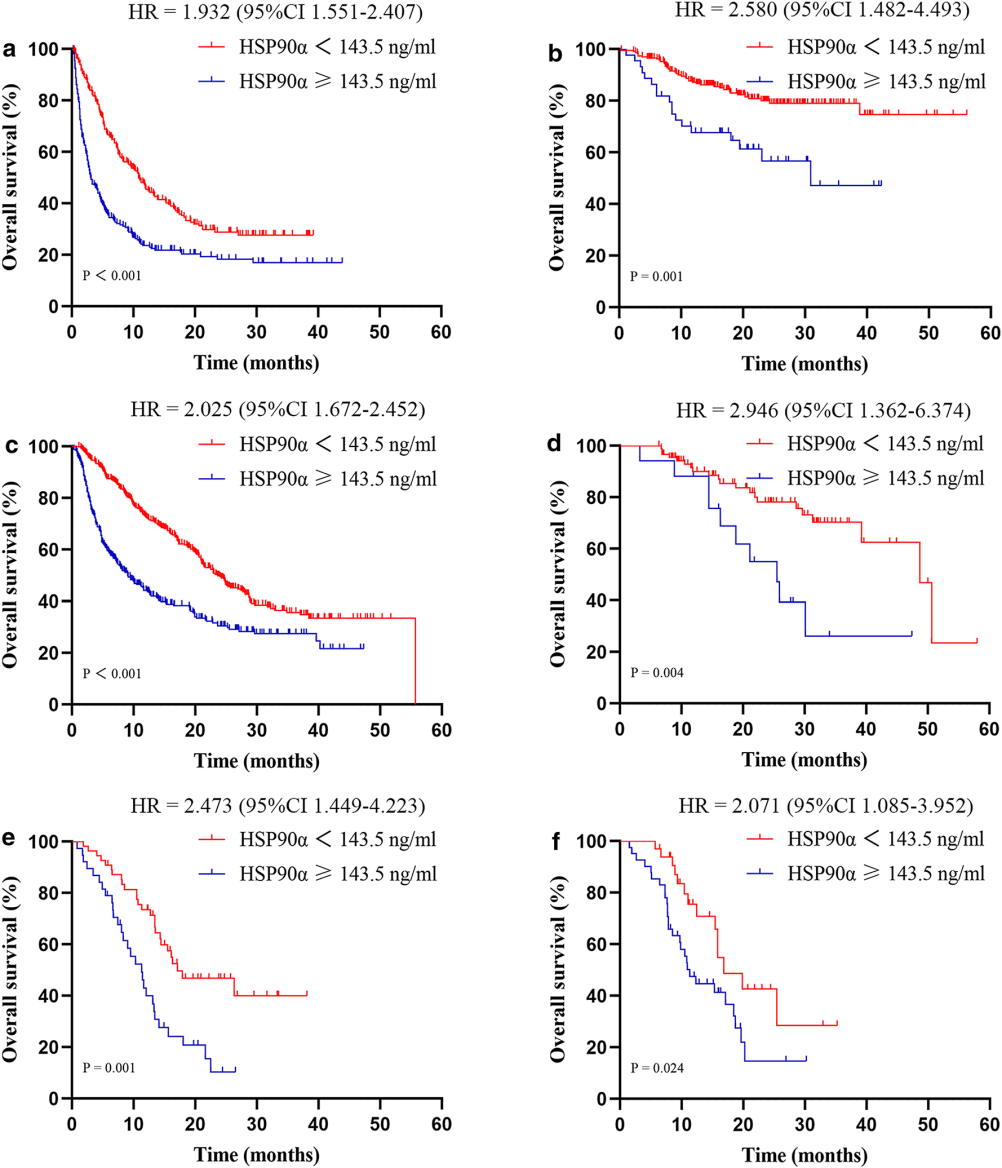
Kaplan–Meier plots for overall survival in the supportive care group (**A**), surgery group (**B**), transcatheter arterial chemoembolization (TACE) group (**C**), adjuvant TACE group (**D**), immune checkpoint inhibitor (ICI) plus targeted therapy group (**E**), and TACE plus ICI group (**F**)

More importantly, in all the six subgroups, patients within the low HSP90α groups consistently demonstrated improved OS compared to the high HSP90α groups (Fig. 3). In univariate and multivariate Cox regression analyses of supportive care, TACE, ICI plus targeted therapy, and TACE plus ICI groups, high HSP90α ≥ 143.5 ng/mL was an independent poor prognostic factor for OS (*p* = 0.006, *p* < 0.001, *p* = 0.047, *p* = 0.027, respectively) (Supplementary Tables 9, 11, 13, 14). Notably, in the surgery and adjuvant TACE group, the HSP90α plasma level was not a significant prognostic factor for OS (Supplementary Tables 10, 12).

### Relationship between HSP90α level and baseline characteristics

**Fig. 4:**
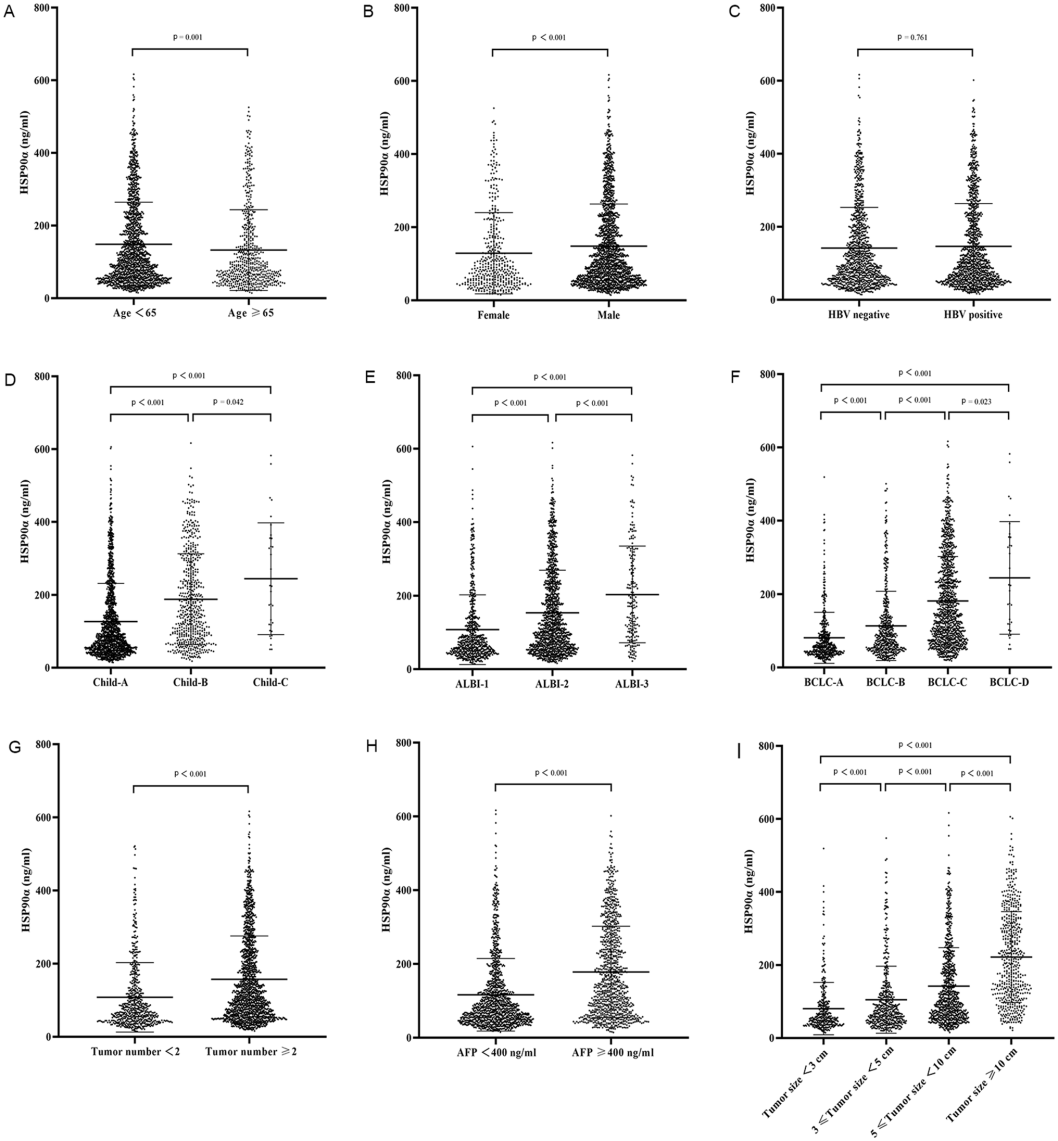
Relationship between the HSP90α levels and baseline characteristics. The HSP90α level was not related to the status of HBV infection (**C**). Higher HSP90α was associated with age ≥ 65 years (**A**), male gender (**B**), worse Child–Pugh grade (**D**), ALBI score (**E**), later BCLC staging (**F**), multiple tumors (**G**), upregulated AFP (**H**), and larger tumor diameter (**I**). *HSP90α* heat-shock protein 90α, *HBV* hepatitis B virus, *ALBI* albumin–bilirubin, *BCLC* Barcelona clinic liver cancer, *AFP* alpha-fetoprotein

Through logistic regression analyses, we confirmed that the age, Child–Pugh class, ALBI grade, AFP, ALP, platelet, ALT, leukocyte, tumor diameter, and PVTT were independent influencing factors for the HSP90α expression (Supplementary Table 15). Moreover, to evaluate the significance of the HSP90α levels in a clinical setting, we further explored the relationship between HSP90α levels and baseline characteristics. The results revealed that the HSP90α level was not related to HBV infection. However, higher HSP90α was associated with older age ≥ 65 (*p* = 0.001), increased AFP ≥ 400 ng/mL (*p* < 0.001), male gender (*p* < 0.001), multiple HCC tumors (*p* < 0.001), more aggressive Child grade (*p* < 0.001) and ALBI score (*p* < 0.001), larger tumor diameter (*p* < 0.001), and more aggressive BCLC staging (*p* < 0.001) (Fig. 4).

## Discussion

HCC is cancer with an aggressive clinical course and high morbidity. Prognostic markers are widely used in clinical practice and have high clinical value as efficient treatment determinants [20]. HSP90α has been previously reported to have a high diagnostic value in patients with HCC [18, 19]. Our novel large-scale, the multicenter study provided robust data on the suitability of the HSP90α plasma level as a prognostic biomarker for HCC. Our results suggested that patients with HSP90α < 143.5 ng/mL had longer mOS compared to patients with HSP90α ≥ 143.5 ng/mL: (*p* < 0.001), implicating that HSP90α ≥ 143.5 ng/mL is an independent poor prognostic factor for OS.

AFP is the most widely used biomarker in HCC to date. Nevertheless, AFP-negative tumors account for about 30% of cases of HCC, with several studies revealing AFP's inability to evaluate this subset of HCC tumors [10, 11, 21]. Therefore, to eradicate this clinical decision gap, new prognostic markers are urgently needed. HSP90α is a master regulator and molecular chaperone regulating key cell signaling networks [22]. The secretion of HSP90α in normal cells promotes tissue repair under stress, while the secretion in tumor cells can promote cancer cell proliferation and metastatic potential [17]. Previous studies have confirmed increased expression of HSP90α levels in several tumor types, including HCC [23, 24]. Furthermore, clinically, HSP90α can be used as a diagnostic biomarker for HCC, lung cancer, breast cancer, and gastric cancer [19, 24–26]. Despite its strong diagnostic value, few studies have elucidated the prognostic value of HSP90α in human cancer. Li et al. reported that lung cancer patients with high HSP90α levels had poorer OS and PFS compared to low HSP90α patients [27]. In addition, a study by Fu et al. found that HSP90α positively correlated with tumor volume after surgery or interventional therapy (*p* < 0.05) [19]. However, the author did not explore the relationship between HSP90α levels with baseline characteristics and patient outcomes. Our study demonstrated that patients with high HSP90α levels had shorter OS and HSP90α was an independent factor for OS in HCC.

Although the protein kinase inhibitor, sorafenib had been used in HCC for many years, its efficacy as monotherapy is still poor, with mOS of only 6.5 months [28]. In recent years, the plethora of studies and drug development advancements of ICIs have expanded our therapeutic arsenal for cancer. The combination of ICIs and targeted drugs has significantly improved the clinical outcomes of HCC patients [29–31]. In the same direction, the combination of TACE plus camrelizumab increased the PFS of advanced HCC patients to 9 months [5]. Nevertheless, predicting the efficacy of HCC patients receiving ICIs remains a clinical challenge with a definite positive outcome in the quality of patient care. In our subgroup analysis (supportive care group, surgery group, TACE group, adjuvant TACE group, ICI plus targeted therapy group, and TACE plus ICI group), all the low HSP90α expressing patient groups demonstrated better OS than the high HSP90α ones. In the multivariate Cox analysis of the supportive care group, TACE group, TACE plus ICI group, and ICI plus targeted therapy group, the HSP90α ≥ 143.5 ng/mL cutoff was also an independent poor prognostic factor for OS. More importantly, in contrast to other more invasive diagnostic techniques, the liquid biopsy technique for the determination of plasma HSP90α levels is characterized by low invasiveness and high convenience. It is a promising, simple, and effective biomarker for assessing survival in HCC patients and discerning the patients who may benefit from specific treatment modalities. Furthermore, our study confirms that HSP90α is associated with prognosis; thus, the follow-up interval should be reduced for HCC patients with a high HSP90α expression. This approach can better predict disease progression and guide in deciding the next treatment strategy. In conclusion, assessing the HSP90α plasma levels is a robust approach to evaluating the treatment efficacy and response of HCC patients.

In our current study, we further explored the relationship between plasma HSP90α levels and baseline clinical characteristics. Strikingly, high HSP90α plasma levels were associated with multiple tumors co-occurrence, worse child grade and ALBI score, larger tumor diameter, and more aggressive BCLC staging. These results further implicate HSP90α as a prognostic factor in HCC. In accordance with our results, recent studies have also demonstrated that high HSP90α levels correlate with a more aggressive clinical stage [18, 19, 24]. Furthermore, our data showed that patients with AFP ≥ 400 ng/mL had higher HSP90α levels compared to patients with AFP < 400 ng/mL. Notably, a study by Xu et al. showed that the HSP90α level detected by immunohistochemistry in HCC tissues did not associate with serum AFP levels [32]. Nevertheless, we interpret these differences based on the fact that HSP90α plasma level determination is a more sensitive method compared to tissue expression via immunohistochemistry.

In addition, our study determined that the high ALP level before and after PSM is an independent negative prognostic factor for OS. Past studies had confirmed that patients with a high ALP expression had a shorter OS than those with a low ALP expression [33–35].

To our knowledge, this is the first comprehensive study with a large sample size to elucidate the association between plasma HSP90α levels and prognosis in HCC patients. As far as the cutoff value is concerned, the value of 143.5 ng/mL was determined as the optimal value by the X-tile software. Subsequently, in our subgroup analysis, we also confirmed that this cutoff value can also be applied as a prognostic and predictive value in different treatment groups. These data have robust clinical significance implicating that the HSP90α plasma level is an important factor to evaluate the therapeutic response of HCC patients in various therapeutic interventions. Despite the advantages of our study, there are still some limitations. First, selection bias cannot be eliminated due to the nature of retrospective studies. Nevertheless, the large sample of our cohort significantly increased the power and robustness of our study. Second, although our study confirmed that the HSP90α level can predict the response of HCC patients to immunotherapy, our results may be affected by the underlying heterogeneity of different ICIs. Future studies with larger cohort samples and classes of ICI should be designed to safely assess these interesting preliminary findings.

## Conclusions

In conclusion, our study confirmed that the plasma HSP90α level can be used as a prognostic and predictive biomarker for HCC. Patients with HSP90α < 143.5 ng/mL had longer mOS compared to those with HSP90α ≥ 143.5 ng/mL. More importantly, HSP90α ≥ 143.5 ng/mL cutoff level was an independent poor prognostic factor for OS in HCC patients. Future prospective studies are required to expand our knowledge on the causal relationship between HSP90α levels and the prognosis of HCC.

## Supplementary Information

Below is the link to the electronic supplementary material.

Supplementary file1 (DOCX 105 KB)

## Data Availability

All data generated or analyzed during this study are included in this article and its supplementary material files. Further inquiries can be directed to the corresponding author (Lanpaoxiansheng @126.com).

## References

[1] Sung H, Ferlay J, Siegel R, Laversanne M, Soerjomataram I, Jemal A (2021). Global cancer statistics 2020: globocan estimates of incidence and mortality worldwide for 36 cancers in 185 countries. CA A Cancer J Clin.

[2] Schöniger-Hekele M, Müller C, Kutilek M, Oesterreicher C, Ferenci P, Gangl AJG (2001). Hepatocellular carcinoma in Central Europe: prognostic features and survival. Gut.

[3] Finn R, Qin S, Ikeda M, Galle P, Ducreux M, Kim T (2020). Atezolizumab plus bevacizumab in unresectable hepatocellular carcinoma. N Engl J Med.

[4] Cheng A, Qin S, Ikeda M, Galle P, Ducreux M, Kim T (2022). Updated efficacy and safety data from IMbrave150: atezolizumab plus bevacizumab vs. sorafenib for unresectable hepatocellular carcinoma. J Hepatol.

[5] Guo Y, Ren Y, Chen L, Sun T, Zhang W, Sun B (2022). Transarterial chemoembolization combined with camrelizumab for recurrent hepatocellular carcinoma. BMC Cancer.

[6] Wang Z, Ren Z, Chen Y, Hu J, Yang G, Yu L (2018). Adjuvant transarterial chemoembolization for HBV-related hepatocellular carcinoma after resection: a randomized controlled study. Clin Cancer Res.

[7] Forner A, Bruix J (2012). Biomarkers for early diagnosis of hepatocellular carcinoma. Lancet Oncol.

[8] Wang N, Cao Y, Song W, He K, Li T, Wang J (2014). Serum peptide pattern that differentially diagnoses hepatitis B virus-related hepatocellular carcinoma from liver cirrhosis. J Gastroenterol Hepatol.

[9] Wang G, Lu X, Du Q, Zhang G, Wang D, Wang Q (2020). Diagnostic value of the γ-glutamyltransferase and alanine transaminase ratio, alpha-fetoprotein, and protein induced by vitamin K absence or antagonist II in hepatitis B virus-related hepatocellular carcinoma. Sci Rep.

[10] Farinati F, Marino D, De Giorgio M, Baldan A, Cantarini M, Cursaro C (2006). Diagnostic and prognostic role of alpha-fetoprotein in hepatocellular carcinoma: both or neither?. Am J Gastroenterol.

[11] Giannini E, Marenco S, Borgonovo G, Savarino V, Farinati F, Del Poggio P (2012). Alpha-fetoprotein has no prognostic role in small hepatocellular carcinoma identified during surveillance in compensated cirrhosis. Hepatology.

[12] Agopian V, Harlander-Locke M, Markovic D, Zarrinpar A, Kaldas F, Cheng E (2017). Evaluation of patients with hepatocellular carcinomas that do not produce α-fetoprotein. JAMA Surg.

[13] Frydman J (2001). Folding of newly translated proteins in vivo: the role of molecular chaperones. Annu Rev Biochem.

[14] Eustace B, Sakurai T, Stewart J, Yimlamai D, Unger C, Zehetmeier C (2004). Functional proteomic screens reveal an essential extracellular role for hsp90 alpha in cancer cell invasiveness. Nat Cell Biol.

[15] Du Y, Wu J, Luo L (2018). Secreted heat shock protein 90α attenuated the effect of anticancer drugs in small-cell lung cancer cells through AKT/GSK3β/β-catenin signaling. Cancer Control.

[16] Zhou X, Wen Y, Tian Y, He M, Ke X, Huang Z (2019). Heat shock protein 90α-dependent B-cell-2-associated transcription factor 1 promotes hepatocellular carcinoma proliferation by regulating MYC proto-oncogene c-MYC mRNA stability. Hepatology.

[17] Wu J, Liu T, Rios Z, Mei Q, Lin X, Cao S (2017). Heat shock proteins and cancer. Trends Pharmacol Sci.

[18] Wei W, Liu M, Ning S, Wei J, Zhong J, Li J (2020). Diagnostic value of plasma HSP90α levels for detection of hepatocellular carcinoma. BMC Cancer.

[19] Fu Y, Xu X, Huang D, Cui D, Liu L, Liu J (2017). Plasma heat shock protein 90alpha as a biomarker for the diagnosis of liver cancer: an official, large-scale, and multicenter clinical trial. EBioMedicine.

[20] Rich N, Murphy C, Yopp A, Tiro J, Marrero J, Singal AG (2020). Sex disparities in presentation and prognosis of 1110 patients with hepatocellular carcinoma. Aliment Pharmacol Ther.

[21] Luo P, Wu S, Yu Y, Ming X, Li S, Zuo X (2020). Current status and perspective biomarkers in AFP negative HCC: towards screening for and diagnosing hepatocellular carcinoma at an earlier stage. Pathol Oncol Res.

[22] El-Serag H, Marrero J, Rudolph L, Reddy KJG (2008). Diagnosis and treatment of hepatocellular carcinoma. Gastroenterology.

[23] Zhou Y, Deng X, Zang N, Li H, Li G, Li C (2015). Transcriptomic and proteomic investigation of HSP90α as a potential biomarker for HCC. Med Sci Monit.

[24] Hou Q, Chen S, An Q, Li B, Fu Y, Luo Y (2021). Extracellular Hsp90α promotes tumor lymphangiogenesis and lymph node metastasis in breast cancer. Int J Mol Sci.

[25] Shi Y, Liu X, Lou J, Han X, Zhang L, Wang Q (2014). Plasma levels of heat shock protein 90 alpha associated with lung cancer development and treatment responses. Clin Cancer Res.

[26] Liang X, Li K, Li Z, Xie M, Tang Y, Du J (2021). Diagnostic and prognostic value of plasma heat shock protein 90alpha in gastric cancer. Int Immunopharmacol.

[27] Li X, Tong X, Liu B, Li Z, Ding J, Li J (2021). Potential predictive value of plasma heat shock protein 90α in lung cancer. J Int Med Res.

[28] Cheng A, Kang Y, Chen Z, Tsao C, Qin S, Kim J (2009). Efficacy and safety of sorafenib in patients in the Asia-Pacific region with advanced hepatocellular carcinoma: a phase III randomised, double-blind, placebo-controlled trial. Lancet Oncol.

[29] Finn R, Ikeda M, Zhu A, Sung M, Baron A, Kudo M (2020). Phase Ib study of lenvatinib plus pembrolizumab in patients with unresectable hepatocellular carcinoma. J Clin Oncol.

[30] Ren Z, Xu J, Bai Y, Xu A, Cang S, Du C (2021). Sintilimab plus a bevacizumab biosimilar (IBI305) versus sorafenib in unresectable hepatocellular carcinoma (ORIENT-32): a randomised, open-label, phase 2–3 study. Lancet Oncol.

[31] Xu J, Shen J, Gu S, Zhang Y, Wu L, Wu J (2021). Camrelizumab in combination with apatinib in patients with advanced hepatocellular carcinoma (RESCUE): a nonrandomized, open-label, phase II trial. Clin Cancer Res.

[32] Xu Q, Tu J, Dou C, Zhang J, Yang L, Liu X (2017). HSP90 promotes cell glycolysis, proliferation and inhibits apoptosis by regulating PKM2 abundance via Thr-328 phosphorylation in hepatocellular carcinoma. Mol Cancer.

[33] Wu S, Lin Y, Ye H, Xiong X, Li F, Cheng NJ (2016). Prognostic value of alkaline phosphatase, gamma-glutamyl transpeptidase and lactate dehydrogenase in hepatocellular carcinoma patients treated with liver resection. Int J Surg.

[34] Fang K, Kao W, Su C, Chen P, Lee P, Huang Y (2018). The prognosis of single large hepatocellular carcinoma was distinct from Barcelona clinic liver cancer stage A or B: the role of albumin-bilirubin grade liver cancer. Liver Cancer.

[35] Llovet J, Singal A, Villanueva A, Finn R, Kudo M, Galle P (2022). Prognostic and predictive factors in patients with advanced HCC and elevated alpha-fetoprotein treated with ramucirumab in two randomized phase III trials. Clin Cancer Res.

