# Supplementary material for: Heat-shock protein 90α is a potential prognostic and predictive biomarker in hepatocellular carcinoma: a large-scale and multicenter study
Source: Hepatol Int. 2022 Aug 16;16(5):1208–19. doi: 10.1007/s12072-022-10391-y (PMC9525341; doi:10.1007/s12072-022-10391-y)
Supplement: Supplementary file 1 — Supplementary file1 (DOCX 105 KB) [file 12072_2022_10391_MOESM1_ESM.docx]

| **Supplementary Table 1. Baseline characteristics after propensity score matching** | | | | |
| --- | --- | --- | --- | --- |
| Variable | Total | HSP90α < 143.5 ng/ml | HSP90α ≥ 143.5 ng/ml | *p* |
| Patients | 970 | 485 | 485 |  |
| Male sex | 800 (82.5) | 401 (82.7) | 399 (82.3) | 0.934 |
| Age ≥ 65 years | 260 (26.8) | 142 (29.3) | 118 (24.3) | 0.098 |
| Etiology |  |  |  |  |
| HBV | 498 (51.3) | 245 (50.5) | 253 (52.2) | 0.650 |
| HCV | 15 (1.5) | 5 (1.0) | 10 (2.1) | 0.302 |
| Alcohol | 412 (42.5) | 209 (43.1) | 203 (41.9) | 0.750 |
| NAFLD | 18 (1.9) | 10 (2.1) | 8 (1.6) | 0.815 |
| Other | 19 (2.0) | 10 (2.1) | 9 (1.9) | 1.000 |
| Diabetes mellitus | 97 (10.0) | 53 (10.9) | 44 (9.1) | 0.391 |
| Hypertension | 145 (14.9) | 80 (16.5) | 65 (13.4) | 0.210 |
| Child–Pugh class |  |  |  | 0.731 |
| A | 670 (69.1) | 333 (68.7) | 337 (69.5) |  |
| B | 285 (29.4) | 143 (29.5) | 142 (29.3) |  |
| C | 15 (1.5) | 9 (1.9) | 6 (1.2) |  |
| ALBI grade |  |  |  | 0.337 |
| 1 | 243 (25.1) | 130 (26.8) | 113 (23.3) |  |
| 2 | 635 (65.5) | 304 (62.7) | 331 (68.2) |  |
| 3 | 92 (9.5) | 51 (10.5) | 41 (8.5) |  |
| HSP90α, median (IQR, ng/ml) | 143.5 (85.3-231.5) | 85.4 (60.5-111.9) | 231 (182.4-310.6) |  |
| Creatinine, median (IQR, mg/dL) | 63.0 (53.8-72.8) | 63.0 (54.6-72.9) | 63.0 (53.0-72.6) | 0.537 |
| Serum AFP, ng/ml |  |  |  | 0.450 |
| < 200 | 496 (51.1) | 251 (51.8) | 245 (50.5) |  |
| ≥ 200, < 400 | 69 (7.1) | 40 (8.2) | 29 (6.0) |  |
| ≥ 400 | 405 (41.8) | 194 (40.0) | 211 (43.5) |  |
| ALP levels ≥ 125 U/L | 650 (67.0) | 325 (67.0) | 325 (67.0) | 1.000 |
| Platelet count ≥ 100 × 109/L | 753 (77.6) | 377 (77.7) | 376 (77.5) | 1.000 |
| ALT levels ≥ 40 U/L | 537 (55.4) | 262 (54) | 275 (56.7) | 0.422 |
| Leukocyte ≥ 4 × 10^9^/L | 853 (87.9) | 427 (88) | 426 (87.8) | 1.000 |
| BCLC stage |  |  |  | 0.464 |
| 0/A | 110 (11.3) | 49 (10.1) | 61 (12.6) |  |
| B | 194 (20.0) | 96 (19.8) | 98 (20.2) |  |
| C | 651 (67.1) | 331 (68.2) | 320 (66.0) |  |
| D | 15 (1.5) | 9 (1.9) | 6 (1.2) |  |
| Number of tumors ≥ 2 | 775 (79.9) | 388 (80) | 387 (79.8) | 1.000 |
| Tumor diameter, cm |  |  |  | 0.222 |
| < 3 | 71 (7.3) | 32 (6.6) | 39 (8.0) |  |
| ≥ 3, < 5 | 151 (15.6) | 77 (15.9) | 74 (15.3) |  |
| ≥ 5, < 10 | 449 (46.3) | 234 (48.2) | 215 (44.3) |  |
| ≥ 10 | 299 (30.8) | 142 (29.3) | 157 (32.4) |  |
| PVTT | 418 (43.1) | 216 (44.5) | 202 (41.6) | 0.356 |
| Lymph node metastasis | 454 (46.8) | 236 (48.7) | 218 (44.9) | 0.238 |
| Extrahepatic metastases | 223 (23.0) | 119 (24.5) | 104 (21.4) | 0.290 |
| Lung | 131 (13.5) | 63 (13.0) | 68 (14.0) |  |
| Bone | 60 (6.2) | 32 (6.6) | 28 (5.8) |  |
| Other | 91 (9.4) | 51 (10.5) | 40 (8.2) |  |
| Treatments |  |  |  |  |
| Supportive care | 221 (22.8) | 103 (21.2) | 118 (24.3) | 0.290 |
| Liver resection | 142 (14.6) | 70 (14.4) | 72 (14.8) | 0.927 |
| Radiotherapy | 28 (2.9) | 16 (3.3) | 12 (2.5) | 0.572 |
| TACE | 537 (55.4) | 272 (56.1) | 265 (54.6) | 0.703 |
| RFA | 19 (2.0) | 8 (1.6) | 11 (2.3) | 0.648 |
| ICI | 117 (12.1) | 65 (13.4) | 52 (10.7) | 0.232 |
| Targeted therapy | 92 (9.5) | 55 (11.3) | 37 (7.6) | 0.060 |
| Chemotherapy | 54 (5.6) | 26 (5.4) | 28 (5.8) | 0.888 |
| Abbreviations: HBV, hepatitis B virus; HCV, hepatitis C virus; NAFLD, nonalcoholic fatty liver disease; ALBI, albumin-bilirubin; HSP90α, heat shock protein 90α; AFP, alpha fetoprotein; ALP, alkaline phosphatase; ALT, alanine aminotransferase; BCLC, Barcelona Clinic Liver Cancer; PVTT, portal vein tumor thrombus; TACE, transcatheter arterial chemoembolization; RFA, radiofrequency ablation; ICI, immune checkpoint inhibitor. | | | | |

| **Supplementary Table 2. Univariate and multivariate Cox regression analysis of overall survival after PSM** | | | | | | | |
| --- | --- | --- | --- | --- | --- | --- | --- |
|  | Univariable Cox regression | | |  | Multivariable Cox regression | | |
|  | HR | 95%CI | *p* |  | HR | 95%CI | *p* |
| Sex (male/female) | 1.144 | 0.919-1.425 | 0.229 |  |  |  |  |
| Age (≥65/<65 years) | 0.949 | 0.787-1.144 | 0.582 |  |  |  |  |
| HBV (positive/negative) | 1.051 | 0.892-1.239 | 0.552 |  |  |  |  |
| HCV (positive/negative) | 1.043 | 0.519-2.096 | 0.906 |  |  |  |  |
| Alcoholism (positive/negative) | 0.991 | 0.839-1.170 | 0.915 |  |  |  |  |
| NAFLD (positive/negative) | 0.678 | 0.337-1.362 | 0.275 |  |  |  |  |
| Diabetes mellitus (positive/negative) | 1.282 | 0.990-1.660 | 0.060 |  |  |  |  |
| Hypertension (positive/negative) | 0.898 | 0.706-1.142 | 0.381 |  |  |  |  |
| Child-Pugh class (B+C/A) | 1.496 | 1.259-1.779 | < 0.001 |  | 1.118 | 0.918-1.362 | 0.268 |
| ALBI grade (2+3/1) | 1.363 | 1.122-1.656 | 0.002 |  | 1.101 | 0.891-1.36 | 0.373 |
| HSP90α (≥ 143.5/< 143.5 ng/ml) | 1.529 | 1.296-1.804 | < 0.001 |  | 1.606 | 1.359-1.898 | < 0.001 |
| AFP (≥400/<400 ng/ml) | 1.069 | 0.907-1.259 | 0.425 |  |  |  |  |
| ALP (≥125/<125 U/L) | 1.615 | 1.346-1.938 | < 0.001 |  | 1.363 | 1.129-1.645 | 0.001 |
| Platelet (<100000/≥100000/μL) | 0.864 | 0.712-1.048 | 0.137 |  |  |  |  |
| ALT (≥40/<40U/L) | 1.082 | 0.917-1.277 | 0.350 |  |  |  |  |
| Leukocyte (<4000/≥4000/μL) | 0.894 | 0.701-1.140 | 0.366 |  |  |  |  |
| BCLC stage |  |  | < 0.001 |  |  |  | 0.315 |
| 0/A | 1.000 |  |  |  | 1.000 |  |  |
| B | 1.678 | 1.170-2.406 | 0.005 |  | 1.302 | 0.831-2.04 | 0.249 |
| C | 2.481 | 1.798-3.423 | < 0.001 |  | 1.511 | 0.95-2.405 | 0.081 |
| D | 4.927 | 2.585-9.390 | < 0.001 |  | 1.705 | 0.837-3.47 | 0.141 |
| Number of tumor (≥2/<2) | 1.771 | 1.406-2.231 | < 0.001 |  | 1.431 | 1.069-1.916 | 0.016 |
| Tumor diameter (≥5/<5 cm) | 0.967 | 0.797-1.172 | 0.731 |  |  |  |  |
| PVTT (positive/negative) | 1.295 | 1.098-1.528 | 0.002 |  | 1.097 | 0.887-1.358 | 0.392 |
| Lymph node metastasis (yes/no) | 1.493 | 1.266-1.760 | < 0.001 |  | 1.048 | 0.846-1.297 | 0.669 |
| Extrahepatic metastases (yes/no) | 1.572 | 1.305-1.894 | < 0.001 |  | 1.255 | 1.016-1.549 | 0.035 |
| Antitumor therapy (no/yes) | 2.476 | 2.064-2.970 | < 0.001 |  | 2.108 | 1.735-2.563 | < 0.001 |
| Abbreviations: PSM, propensity score matching; HBV, hepatitis B virus; HCV, hepatitis C virus; NAFLD, nonalcoholic fatty liver disease; ALBI, albumin-bilirubin; HSP90α, heat shock protein 90α; AFP, alpha fetoprotein; ALP, alkaline phosphatase; ALT, alanine aminotransferase; BCLC, Barcelona Clinic Liver Cancer; PVTT, portal vein tumor thrombus. | | | | | | | |

| **Supplementary Table 3. Baseline characteristics of supportive care group** | | | |  |
| --- | --- | --- | --- | --- |
| Variable | Total | HSP90α < 143.5 ng/ml | HSP90α ≥ 143.5 ng/ml | *p* |
| Patients | 457 | 232 | 225 |  |
| Male sex | 363 (79.4) | 177 (76.3) | 186 (82.7) | 0.092 |
| Age ≥ 65 years | 155 (33.9) | 87 (37.5) | 68 (30.2) | 0.100 |
| Etiology |  |  |  |  |
| HBV | 199 (43.5) | 90 (38.8) | 109 (48.4) | 0.037 |
| HCV | 6 (1.3) | 2 (0.9) | 4 (1.8) | 0.390 |
| Alcohol | 193 (42.2) | 94 (40.5) | 99 (44) | 0.451 |
| NAFLD | 5 (1.1) | 4 (1.7) | 1 (0.4) | 0.189 |
| Other | 6 (1.3) | 3 (1.3) | 3 (1.3) | 0.970 |
| Diabetes mellitus | 45 (9.8) | 24 (10.3) | 21 (9.3) | 0.717 |
| Hypertension | 67 (14.7) | 38 (16.4) | 29 (12.9) | 0.292 |
| Child–Pugh class |  |  |  | < 0.001 |
| A | 231 (50.5) | 150 (64.7) | 81 (36) |  |
| B | 194 (42.5) | 70 (30.2) | 124 (55.1) |  |
| C | 32 (7.0) | 12 (5.2) | 20 (8.9) |  |
| ALBI grade |  |  |  | < 0.001 |
| 1 | 91 (19.9) | 71 (30.6) | 20 (8.9) |  |
| 2 | 252 (55.1) | 111 (47.8) | 141 (62.7) |  |
| 3 | 114 (24.9) | 50 (21.6) | 64 (28.4) |  |
| HSP90α, median (IQR, ng/ml) | 140.2 (75.2-269.5) | 75.8 (52.2-102.4) | 270.4 (208.1-365.3) | 0.732 |
| Creatinine, median (IQR, mg/dL) | 64.0 (55.0-74.8) | 64.8 (55.0-74.3) | 64.0 (54.9-76.0) |  |
| Serum AFP, ng/ml |  |  |  | < 0.001 |
| < 200 | 254 (55.6) | 166 (71.6) | 88 (39.1) |  |
| ≥ 200, < 400 | 32 (7.0) | 17 (7.3) | 15 (6.7) |  |
| ≥ 400 | 171 (37.4) | 49 (21.1) | 122 (54.2) |  |
| ALP levels ≥ 125 U/L | 328 (71.8) | 134 (57.8) | 194 (86.2) | < 0.001 |
| Platelet count ≥ 100 × 109/L | 351 (76.8) | 176 (75.9) | 175 (77.8) | 0.628 |
| ALT levels ≥ 40 U/L | 275 (60.2) | 121 (52.2) | 154 (68.4) | < 0.001 |
| Leukocyte ≥ 4 × 10^9^/L | 393 (86.0) | 187 (80.6) | 206 (91.6) | 0.001 |
| BCLC stage |  |  |  | < 0.001 |
| 0/A | 48 (10.5) | 39 (16.8) | 9 (4.0) |  |
| B | 54 (11.8) | 43 (18.5) | 11 (4.9) |  |
| C | 323 (70.7) | 138 (59.5) | 185 (82.2) |  |
| D | 32 (7.0) | 12 (5.2) | 20 (8.9) |  |
| Number of tumors ≥ 2 | 363 (79.4) | 168 (72.4) | 195 (86.7) | < 0.001 |
| Tumor diameter, cm |  |  |  | < 0.001 |
| < 3 | 57 (12.5) | 43 (18.5) | 14 (6.2) |  |
| ≥ 3, < 5 | 101 (22.1) | 73 (31.5) | 28 (12.4) |  |
| ≥ 5, < 10 | 180 (39.4) | 86 (37.1) | 94 (41.8) |  |
| ≥ 10 | 119 (26) | 30 (12.9) | 89 (39.6) |  |
| PVTT | 184 (40.3) | 56 (24.1) | 128 (56.9) | < 0.001 |
| Lymph node metastasis | 266 (58.2) | 113 (48.7) | 153 (68.0) | < 0.001 |
| Extrahepatic metastases | 144 (31.5) | 63 (27.2) | 81 (36.0) | 0.042 |
| Lung | 92 (20.1) | 34 (14.7) | 58 (25.8) |  |
| Bone | 30 (6.6) | 15 (6.5) | 15 (6.7) |  |
| Other | 65 (14.2) | 29 (12.5) | 36 (16.0) |  |
| Abbreviations: HBV, hepatitis B virus; HCV, hepatitis C virus; NAFLD, nonalcoholic fatty liver disease; ALBI, albumin-bilirubin; HSP90α, heat shock protein 90α; AFP, alpha fetoprotein; ALP, alkaline phosphatase; ALT, alanine aminotransferase; BCLC, Barcelona Clinic Liver Cancer; PVTT, portal vein tumor thrombus. | | | | |

| **Supplementary Table 4. Baseline characteristics of surgical group** | | | | | | | | |  |
| --- | --- | --- | --- | --- | --- | --- | --- | --- | --- |
| Variable | | Total | | HSP90α < 143.5 ng/ml | | HSP90α ≥ 143.5 ng/ml | | | *p* |
| Patients | | 275 | | 231 | | 44 | | |  |
| Male sex | | 200 (72.7) | | 166 (71.9) | | 34 (77.3) | | | 0.460 |
| Age ≥ 65 years | | 85 (30.9) | | 69 (29.9) | | 16 (36.4) | | | 0.393 |
| Etiology | |  | |  | |  | | |  |
| HBV | | 150 (54.5) | | 128 (55.4) | | 22 (50.0) | | | 0.509 |
| HCV | | 4 (1.5) | | 3 (1.3) | | 1 (2.3) | | | 0.621 |
| Alcohol | | 101 (36.7) | | 79 (34.2) | | 22 (50.0) | | | 0.046 |
| NAFLD | | 3 (1.1) | | 2 (0.9) | | 1 (2.3) | | | 0.410 |
| Other | | 2 (0.7) | | 1 (0.4) | | 1 (2.3) | | | 0.188 |
| Diabetes mellitus | | 32 (11.6) | | 26 (11.3) | | 6 (13.6) | | | 0.652 |
| Hypertension | | 54 (19.6) | | 44 (19.0) | | 10 (22.7) | | | 0.573 |
| Child–Pugh class | |  | |  | |  | | | 0.050 |
| A | | 233 (84.7) | | 200 (86.6) | | 33 (75.0) | | |  |
| B | | 42 (15.3) | | 31 (13.4) | | 11 (25.0) | | |  |
| ALBI grade | |  | |  | |  | | | < 0.001 |
| 1 | | 108 (39.3) | | 102 (44.2) | | 6 (13.6) | | |  |
| 2 | | 152 (55.3) | | 119 (51.5) | | 33 (75.0) | | |  |
| 3 | | 15 (5.5) | | 10 (4.3) | | 5 (11.4) | | |  |
| HSP90α, median (IQR, ng/ml) | | 63.1 (42.9-118.7) | | 56.2 (40.6-88.4) | | 198.7 (172.3-299.1) | | |  |
| Creatinine, median (IQR, mg/dL) | | 63.6 (54.5-74.5) | | 65.7 (55.0-75.6) | | 59.5 (52.3-65.1) | | | 0.051 |
| Serum AFP, ng/ml | |  | |  | |  | | | 0.058 |
| < 200 | | 176 (64.0) | | 154 (66.7) | | 22 (50.0) | | |  |
| ≥ 200, < 400 | | 12 (4.4) | | 8 (3.5) | | 4 (9.1) | | |  |
| ≥ 400 | | 87 (31.6) | | 69 (29.9) | | 18 (40.9) | | |  |
| ALP levels ≥ 125 U/L | | 92 (33.5) | | 67 (29.0) | | 25 (56.8) | | | < 0.001 |
| Platelet count ≥ 100 × 109/L | | 213 (77.5) | | 178 (77.1) | | 35 (79.5) | | | 0.717 |
| ALT levels ≥ 40 U/L | | 130 (47.3) | | 103 (44.6) | | 27 (61.4) | | | 0.041 |
| Leukocyte ≥ 4 × 10^9^/L | | 233 (84.7) | | 191 (82.7) | | 42 (95.5) | | | 0.031 |
| BCLC stage | |  | |  | |  | | | < 0.001 |
| 0/A | | 155 (56.4) | | 141 (61.0) | | 14 (31.8) | | |  |
| B | | 80 (29.1) | | 63 (27.3) | | 17 (38.6) | | |  |
| C | | 40 (14.5) | | 27 (11.7) | | 13 (29.5) | | |  |
| Number of tumors ≥ 2 | | 130 (47.3) | | 105 (45.5) | | 25 (56.8) | | | 0.166 |
| Tumor diameter, cm | |  | |  | |  | | | < 0.001 |
| < 3 | | 79 (28.7) | | 74 (32) | | 5 (11.4) | | |  |
| ≥ 3, < 5 | | 67 (24.4) | | 58 (25.1) | | 9 (20.5) | | |  |
| ≥ 5, < 10 | | 101 (36.7) | | 83 (35.9) | | 18 (40.9) | | |  |
| ≥ 10 | | 28 (10.2) | | 16 (6.9) | | 12 (27.3) | | |  |
| PVTT | | 40 (14.5) | | 27 (11.7) | | 13 (29.5) | | | 0.002 |
| Abbreviations: HBV, hepatitis B virus; HCV, hepatitis C virus; NAFLD, nonalcoholic fatty liver disease; ALBI, albumin-bilirubin; HSP90α, heat shock protein 90α; AFP, alpha fetoprotein; ALP, alkaline phosphatase; ALT, alanine aminotransferase; BCLC, Barcelona Clinic Liver Cancer; PVTT, portal vein tumor thrombus. | | | | | | | | | |
| **Supplementary Table 5. Baseline characteristics of TACE group** | | | | | | |  |  |  |
| Variable | Total | | HSP90α < 143.5 ng/ml | | HSP90α ≥ 143.5 ng/ml | | *p* |  |  |
| Patients | 780 | | 452 | | 328 | |  |  |  |
| Male sex | 660 (84.6) | | 375 (83.0) | | 285 (86.9) | | 0.134 |  |  |
| Age ≥ 65 years | 192 (24.6) | | 133 (29.4) | | 59 (18.0) | | < 0.001 |  |  |
| Etiology |  | |  | |  | |  |  |  |
| HBV | 468 (60.0) | | 275 (60.8) | | 193 (58.8) | | 0.574 |  |  |
| HCV | 24 (3.1) | | 16 (3.5) | | 8 (2.4) | | 0.380 |  |  |
| Alcohol | 337 (43.2) | | 190 (42) | | 147 (44.8) | | 0.439 |  |  |
| NAFLD | 10 (1.3) | | 6 (1.3) | | 4 (1.2) | | 0.895 |  |  |
| Other | 14 (1.8) | | 9 (2.0) | | 5 (1.5) | | 0.628 |  |  |
| Diabetes mellitus | 65 (8.3) | | 41 (9.1) | | 24 (7.3) | | 0.382 |  |  |
| Hypertension | 108 (13.8) | | 68 (15) | | 40 (12.2) | | 0.255 |  |  |
| Child–Pugh class |  | |  | |  | | < 0.001 |  |  |
| A | 581 (74.5) | | 363 (80.3) | | 218 (66.5) | |  |  |  |
| B | 199 (25.5) | | 89 (19.7) | | 110 (33.5) | |  |  |  |
| ALBI grade |  | |  | |  | | < 0.001 |  |  |
| 1 | 205 (26.3) | | 145 (32.1) | | 60 (18.3) | |  |  |  |
| 2 | 535 (68.6) | | 295 (65.3) | | 240 (73.2) | |  |  |  |
| 3 | 40 (5.1) | | 12 (2.7) | | 28 (8.5) | |  |  |  |
| HSP90α, median (IQR, ng/ml) | 121.4 (65.3-227.1) | | 72.0 (48.2-104.3) | | 258.2 (188.4-329.7) | |  |  |  |
| Creatinine, median (IQR, mg/dL) | 63.4 (54.4-72.1) | | 63.4 (54.8-73.0) | | 63.5 (54.0-72.0) | | 0.340 |  |  |
| Serum AFP, ng/ml |  | |  | |  | | < 0.001 |  |  |
| < 200 | 358 (45.9) | | 245 (54.2) | | 113 (34.5) | |  |  |  |
| ≥ 200, < 400 | 54 (6.9) | | 37 (8.2) | | 17 (5.2) | |  |  |  |
| ≥ 400 | 368 (47.2) | | 170 (37.6) | | 198 (60.4) | |  |  |  |
| ALP levels ≥ 125 U/L | 469 (60.1) | | 221 (48.9) | | 248 (75.6) | | < 0.001 |  |  |
| Platelet count ≥ 100 × 109/L | 547 (70.1) | | 285 (63.1) | | 262 (79.9) | | < 0.001 |  |  |
| ALT levels ≥ 40 U/L | 418 (53.6) | | 206 (45.6) | | 212 (64.6) | | < 0.001 |  |  |
| Leukocyte ≥ 4 × 10^9^/L | 629 (80.6) | | 340 (75.2) | | 289 (88.1) | | < 0.001 |  |  |
| BCLC stage |  | |  | |  | | < 0.001 |  |  |
| 0/A | 101 (12.9) | | 82 (18.1) | | 19 (5.8) | |  |  |  |
| B | 199 (25.5) | | 145 (32.1) | | 54 (16.5) | |  |  |  |
| C | 480 (61.5) | | 225 (49.8) | | 255 (77.7) | |  |  |  |
| Number of tumors ≥ 2 | 629 (80.6) | | 350 (77.4) | | 279 (85.1) | | 0.008 |  |  |
| Tumor diameter, cm |  | |  | |  | | < 0.001 |  |  |
| < 3 | 79 (10.1) | | 65 (14.4) | | 14 (4.3) | |  |  |  |
| ≥ 3, < 5 | 135 (17.3) | | 105 (23.2) | | 30 (9.1) | |  |  |  |
| ≥ 5, < 10 | 303 (38.8) | | 186 (41.2) | | 117 (35.7) | |  |  |  |
| ≥ 10 | 263 (33.7) | | 96 (21.2) | | 167 (50.9) | |  |  |  |
| PVTT | 321 (41.2) | | 133 (29.4) | | 188 (57.3) | | < 0.001 |  |  |
| Lymph node metastasis | 351 (45.0) | | 161 (35.6) | | 190 (57.9) | | < 0.001 |  |  |
| Extrahepatic metastases | 138 (17.7) | | 62 (13.7) | | 76 (23.2) | | 0.001 |  |  |
| Lung | 87 (11.2) | | 35 (7.7) | | 52 (15.9) | |  |  |  |
| Bone | 29 (3.7) | | 15 (3.3) | | 14 (4.3) | |  |  |  |
| Other | 39 (5.0) | | 21 (4.6) | | 18 (5.5) | |  |  |  |
| Abbreviations: HBV, hepatitis B virus; HCV, hepatitis C virus; NAFLD, nonalcoholic fatty liver disease; ALBI, albumin-bilirubin; HSP90α, heat shock protein 90α; AFP, alpha fetoprotein; ALP, alkaline phosphatase; ALT, alanine aminotransferase; BCLC, Barcelona Clinic Liver Cancer; PVTT, portal vein tumor thrombus; TACE, transcatheter arterial chemoembolization. | | | | | | | |  |  |

| **Supplementary Table 6. Baseline characteristics of adjuvant TACE group** | | | |  |
| --- | --- | --- | --- | --- |
| Variable | Total | HSP90α < 143.5 ng/ml | HSP90α ≥ 143.5 ng/ml | *p* |
| Patients | 107 | 90 | 17 |  |
| Male sex | 88 (82.2) | 73 (81.1) | 15 (88.2) | 0.481 |
| Age ≥ 65 years | 26 (24.3) | 23 (25.6) | 3 (17.6) | 0.486 |
| Etiology |  |  |  |  |
| HBV | 71 (66.4) | 64 (71.1) | 7 (41.2) | 0.017 |
| HCV | 2 (1.9) | 2 (2.2) | 0 | 0.535 |
| Alcohol | 44 (41.1) | 37 (41.1) | 7 (41.2) | 0.996 |
| NAFLD | 3 (2.8) | 2 (2.2) | 1 (5.9) | 0.402 |
| Other | 3 (2.8) | 2 (2.2) | 1 (5.9) | 0.402 |
| Diabetes mellitus | 8 (7.5) | 7 (7.8) | 1 (5.9) | 0.785 |
| Hypertension | 23 (21.5) | 21 (23.3) | 2 (11.8) | 0.287 |
| Child–Pugh class |  |  |  | 0.593 |
| A | 97 (90.7) | 81 (90.0) | 16 (94.1) |  |
| B | 10 (9.3) | 9 (10.0) | 1 (5.9) |  |
| ALBI grade |  |  |  | 0.848 |
| 1 | 58 (54.2) | 48 (53.3) | 10 (58.8) |  |
| 2 | 48 (44.9) | 41 (45.6) | 7 (41.2) |  |
| 3 | 1 (0.9) | 1 (1.1) | 0 |  |
| HSP90α, median (IQR, ng/ml) | 58.1 (42.9-91.4) | 51.5 (39.5-79.3) | 250.3 (197.1-326.8) |  |
| Creatinine, median (IQR, mg/dL) | 67.9 (58.0-76.0) | 67.4 (58.0-75.4) | 71.6 (55.9-82.7) | 0.618 |
| Serum AFP, ng/ml |  |  |  | 0.709 |
| < 200 | 71 (66.4) | 61 (67.8) | 10 (58.8) |  |
| ≥ 200, < 400 | 7 (6.5) | 6 (6.7) | 1 (5.9) |  |
| ≥ 400 | 29 (27.1) | 23 (25.6) | 6 (35.3) |  |
| ALP levels ≥ 125 U/L | 24 (22.4) | 17 (18.9) | 7 (41.2) | 0.043 |
| Platelet count ≥ 100 × 109/L | 77 (72) | 66 (73.3) | 11 (64.7) | 0.468 |
| ALT levels ≥ 40 U/L | 41 (38.3) | 33 (36.7) | 8 (47.1) | 0.419 |
| Leukocyte ≥ 4 × 10^9^/L | 93 (86.9) | 78 (86.7) | 15 (88.2) | 0.86 |
| BCLC stage |  |  |  | 0.002 |
| 0/A | 43 (40.2) | 37 (41.1) | 6 (35.3) |  |
| B | 52 (48.6) | 47 (52.2) | 5 (29.4) |  |
| C | 12 (11.2) | 6 (6.7) | 6 (35.3) |  |
| Number of tumors ≥ 2 | 64 (59.8) | 56 (62.2) | 8 (47.1) | 0.242 |
| Tumor diameter, cm |  |  |  | < 0.001 |
| < 3 | 16 (15.0) | 16 (17.8) | 0 |  |
| ≥ 3, < 5 | 36 (33.6) | 33 (36.7) | 3 (17.6) |  |
| ≥ 5, < 10 | 43 (40.2) | 39 (43.3) | 4 (23.5) |  |
| ≥ 10 | 12 (11.2) | 2 (2.2) | 10 (58.8) |  |
| PVTT | 12 (11.2) | 6 (6.7) | 6 (35.3) | 0.001 |
| Abbreviations: HBV, hepatitis B virus; HCV, hepatitis C virus; NAFLD, nonalcoholic fatty liver disease; ALBI, albumin-bilirubin; HSP90α, heat shock protein 90α; AFP, alpha fetoprotein; ALP, alkaline phosphatase; ALT, alanine aminotransferase; BCLC, Barcelona Clinic Liver Cancer; PVTT, portal vein tumor thrombus; TACE, transcatheter arterial chemoembolization. | | | | |

| **Supplementary Table 7. Baseline characteristics of ICI plus targeted therapy group** | | | | |
| --- | --- | --- | --- | --- |
| Variable | Total | HSP90α < 143.5 ng/ml | HSP90α ≥ 143.5 ng/ml | *p* |
| Patients | 93 | 55 | 38 |  |
| Male sex | 69 (74.2) | 41 (74.5) | 28 (73.7) | 0.926 |
| Age ≥ 65 years | 20 (21.5) | 17 (30.9) | 3 (7.9) | 0.008 |
| Etiology |  |  |  |  |
| HBV | 49 (52.7) | 29 (52.7) | 20 (52.6) | 0.993 |
| HCV | 1 (1.1) | 0 | 1 (2.6) | 0.226 |
| Alcohol | 33 (35.5) | 20 (36.4) | 13 (34.2) | 0.831 |
| NAFLD | 3 (3.2) | 0 | 3 (7.9) | 0.034 |
| Other | 4 (4.3) | 3 (5.5) | 1 (2.6) | 0.059 |
| Diabetes mellitus | 13 (14) | 9 (16.4) | 4 (10.5) | 0.425 |
| Hypertension | 15 (16.1) | 9 (16.4) | 6 (15.8) | 0.941 |
| Child–Pugh class |  |  |  | 0.107 |
| A | 58 (62.4) | 38 (69.1) | 20 (52.6) |  |
| B | 35 (37.6) | 17 (30.9) | 18 (47.4) |  |
| ALBI grade |  |  |  | 0.197 |
| 1 | 22 (23.7) | 16 (29.1) | 6 (15.8) |  |
| 2 | 59 (63.4) | 34 (61.8) | 25 (65.8) |  |
| 3 | 12 (12.9) | 5 (9.1) | 7 (18.4) |  |
| HSP90α, median (IQR, ng/ml) | 107.6 (65.4-222.5) | 71.8 (57.7-90.3) | 245.8 (184.1-296.2) |  |
| Creatinine, median (IQR, mg/dL) | 60.3 (51.0-73.5) | 62.2 (52.9-73.7) | 58.0 (45.1-73.2) | 0.164 |
| Serum AFP, ng/ml |  |  |  | 0.002 |
| < 200 | 53 (57) | 39 (70.9) | 14 (36.8) |  |
| ≥ 200, < 400 | 4 (4.3) | 3 (5.5) | 1 (2.6) |  |
| ≥ 400 | 36 (38.7) | 13 (23.6) | 23 (60.5) |  |
| ALP levels ≥ 125 U/L | 60 (64.5) | 28 (50.9) | 32 (84.2) | 0.001 |
| Platelet count ≥ 100 × 109/L | 69 (74.2) | 37 (67.3) | 32 (84.2) | 0.067 |
| ALT levels ≥ 40 U/L | 44 (47.3) | 20 (36.4) | 24 (63.2) | 0.011 |
| Leukocyte ≥ 4 × 10^9^/L | 81 (87.1) | 46 (83.6) | 35 (92.1) | 0.231 |
| BCLC stage |  |  |  | 0.201 |
| 0/A | 9 (9.7) | 7 (12.7) | 2 (5.3) |  |
| B | 9 (9.7) | 7 (12.7) | 2 (5.3) |  |
| C | 75 (80.6) | 41 (74.5) | 34 (89.5) |  |
| Number of tumors ≥ 2 | 73 (78.5) | 42 (76.4) | 31 (81.6) | 0.547 |
| Tumor diameter, cm |  |  |  | 0.104 |
| < 3 | 10 (10.8) | 8 (14.5) | 2 (5.3) |  |
| ≥ 3, < 5 | 18 (19.4) | 12 (21.8) | 6 (15.8) |  |
| ≥ 5, < 10 | 42 (45.2) | 26 (47.3) | 16 (42.1) |  |
| ≥ 10 | 23 (24.7) | 9 (16.4) | 14 (36.8) |  |
| PVTT | 47 (50.5) | 18 (32.7) | 29 (76.3) | < 0.001 |
| Lymph node metastasis | 58 (62.4) | 32 (58.2) | 26 (68.4) | 0.316 |
| Extrahepatic metastases | 41 (44.1) | 21 (38.2) | 20 (52.6) | 0.168 |
| Lung | 23 (24.7) | 8 (14.5) | 15 (39.5) |  |
| Bone | 12 (12.9) | 7 (12.7) | 5 (13.2) |  |
| Other | 18 (19.4) | 11 (20) | 7 (18.4) |  |
| Abbreviations: HBV, hepatitis B virus; HCV, hepatitis C virus; NAFLD, nonalcoholic fatty liver disease; ALBI, albumin-bilirubin; HSP90α, heat shock protein 90α; AFP, alpha fetoprotein; ALP, alkaline phosphatase; ALT, alanine aminotransferase; BCLC, Barcelona Clinic Liver Cancer; PVTT, portal vein tumor thrombus; ICI, immune checkpoint inhibitor. | | | | |

| **Supplementary Table 8. Baseline characteristics of TACE plus ICI group** | | | |  |
| --- | --- | --- | --- | --- |
| Variable | Total | HSP90α < 143.5 ng/ml | HSP90α ≥ 143.5 ng/ml | *p* |
| Patients | 74 | 33 | 41 |  |
| Male sex | 61 (82.4) | 27 (81.8) | 34 (82.9) | 0.901 |
| Age ≥ 65 years | 12 (16.2) | 6 (14.6) | 6 (18.2) | 0.681 |
| Etiology |  |  |  |  |
| HBV | 56 (75.7) | 27 (81.8) | 29 (70.7) | 0.269 |
| Alcohol | 34 (45.9) | 14 (42.4) | 20 (48.8) | 0.585 |
| Other | 3 (4.1) | 0 | 3 (7.3) | 0.113 |
| Diabetes mellitus | 6 (8.1) | 5 (15.2) | 1 (2.4) | 0.046 |
| Hypertension | 9 (12.2) | 7 (21.2) | 2 (4.9) | 0.033 |
| Child–Pugh class |  |  |  | 0.225 |
| A | 58 (78.4) | 28 (84.8) | 30 (73.2) |  |
| B | 16 (21.6) | 5 (15.2) | 11 (26.8) |  |
| ALBI grade |  |  |  | 0.263 |
| 1 | 18 (24.3) | 11 (33.3) | 7 (17.1) |  |
| 2 | 53 (71.6) | 21 (63.6) | 32 (78.0) |  |
| 3 | 3 (4.1) | 1 (3.0) | 2 (4.9) |  |
| HSP90α, median (IQR, ng/ml) | 156.0(72.6-257.0) | 69.8(50.0-102.7) | 239.6(173.8-318.8) |  |
| Creatinine, median (IQR, mg/dL) | 59.5(51.7-69.3) | 60.0(53.0-69.6) | 58.0(49.0-69.5) | 0.257 |
| Serum AFP, ng/ml |  |  |  | 0.137 |
| < 200 | 32 (43.2) | 17 (51.5) | 15 (36.6) |  |
| ≥ 200, < 400 | 6 (8.1) | 4 (12.1) | 2 (4.9) |  |
| ≥ 400 | 36 (48.6) | 12 (36.4) | 24 (58.5) |  |
| ALP levels ≥ 125 U/L | 42 (56.8) | 12 (36.4) | 30 (73.2) | 0.001 |
| Platelet count ≥ 100 × 109/L | 61 (82.4) | 25 (75.8) | 36 (87.8) | 0.176 |
| ALT levels ≥ 40 U/L | 35 (47.3) | 13 (39.4) | 22 (53.7) | 0.222 |
| Leukocyte ≥ 4 × 10^9^/L | 67 (90.5) | 29 (87.9) | 38 (92.7) | 0.483 |
| BCLC stage |  |  |  | 0.020 |
| 0/A | 3 (4.1) | 3 (9.1) | 0 |  |
| B | 8 (10.8) | 6 (18.2) | 2 (4.9) |  |
| C | 63 (85.1) | 24 (72.7) | 39 (95.1) |  |
| Number of tumors ≥ 2 | 66 (89.2) | 29 (87.9) | 37 (90.2) | 0.745 |
| Tumor diameter, cm |  |  |  | 0.008 |
| < 3 | 2 (2.7) | 2 (6.1) | 0 |  |
| ≥ 3, < 5 | 9 (12.2) | 8 (24.2) | 1 (2.4) |  |
| ≥ 5, < 10 | 29 (39.2) | 12 (36.4) | 17 (41.5) |  |
| ≥ 10 | 34 (45.9) | 11 (33.3) | 23 (56.1) |  |
| PVTT | 43 (58.1) | 13 (39.4) | 30 (73.2) | 0.003 |
| Lymph node metastasis | 49 (66.2) | 20 (60.6) | 29 (70.7) | 0.360 |
| Extrahepatic metastases | 24 (32.4) | 7 (21.2) | 17 (41.5) | 0.064 |
| Lung | 14 (18.9) | 4 (12.1) | 10 (24.4) |  |
| Bone | 5 (6.8) | 1 (3.0) | 4 (9.8) |  |
| Other | 7 (9.5) | 2 (6.1) | 5 (12.2) |  |
| Abbreviations: HBV, hepatitis B virus; HCV, hepatitis C virus; ALBI, albumin-bilirubin; HSP90α, heat shock protein 90α; AFP, alpha fetoprotein; ALP, alkaline phosphatase; ALT, alanine aminotransferase; BCLC, Barcelona Clinic Liver Cancer; PVTT, portal vein tumor thrombus; TACE, transcatheter arterial chemoembolization; ICI, immune checkpoint inhibitor. | | | | |

| **Supplementary Table 9. Univariate and multivariate Cox regression analysis of overall survival in supportive care group** | | | | | | | |
| --- | --- | --- | --- | --- | --- | --- | --- |
|  | Univariable Cox regression | | |  | Multivariable Cox regression | | |
| Variable | HR | 95%CI | *p* |  | HR | 95%CI | *p* |
| Sex (male/female) | 1.138 | 0.868-1.491 | 0.349 |  |  |  |  |
| Age (≥65/<65 years) | 1.103 | 0.879-1.385 | 0.398 |  |  |  |  |
| HBV (positive/negative) | 0.999 | 0.802-1.245 | 0.996 |  |  |  |  |
| HCV (positive/negative) | 0.841 | 0.314-2.257 | 0.732 |  |  |  |  |
| Alcoholism (positive/negative) | 1.040 | 0.834-1.296 | 0.728 |  |  |  |  |
| Diabetes mellitus (positive/negative) | 1.103 | 0.770-1.581 | 0.593 |  |  |  |  |
| Hypertension (positive/negative) | 0.954 | 0.694-1.312 | 0.773 |  |  |  |  |
| Child-Pugh class (B/A) | 1.492 | 1.200-1.856 | < 0.001 |  | 0.986 | 0.754-1.289 | 0.918 |
| ALBI grade (2+3/1) | 1.755 | 1.317-2.339 | < 0.001 |  | 1.380 | 0.992-1.920 | 0.056 |
| HSP90α (≥ 143.5/< 143.5 ng/ml) | 1.932 | 1.551-2.407 | < 0.001 |  | 1.445 | 1.110-1.881 | 0.006 |
| AFP (≥400/<400 ng/ml) | 1.374 | 1.105-1.709 | 0.004 |  | 1.144 | 0.906-1.444 | 0.258 |
| ALP (≥125/<125 U/L) | 1.847 | 1.430-2.384 | < 0.001 |  | 1.399 | 1.054-1.858 | 0.020 |
| Platelet (<100000/≥100000/μL) | 1.109 | 0.857-1.436 | 0.432 |  |  |  |  |
| ALT (≥40/<40U/L) | 1.103 | 0.883-1.379 | 0.387 |  |  |  |  |
| Leukocyte (<4000/≥4000/μL) | 1.107 | 0.815-1.504 | 0.516 |  |  |  |  |
| BCLC stage |  |  | 0.001 |  |  |  | 0.335 |
| 0/A | 1.000 |  |  |  | 1.000 |  |  |
| B | 0.660 | 0.406-1.071 | 0.092 |  | 0.679 | 0.415-1.109 | 0.122 |
| C | 1.344 | 0.942-1.919 | 0.103 |  | 1.006 | 0.637-1.589 | 0.980 |
| D | 1.460 | 0.873-2.439 | 0.149 |  | 0.915 | 0.513-1.633 | 0.763 |
| Number of tumor (≥2/<2) | 1.118 | 0.854-1.463 | 0.416 |  |  |  |  |
| Tumor diameter (≥5/<5 cm) | 1.419 | 1.126-1.788 | 0.003 |  | 1.101 | 0.851-1.424 | 0.465 |
| PVTT (positive/negative) | 1.549 | 1.241-1.933 | < 0.001 |  | 1.051 | 0.808-1.366 | 0.712 |
| Lymph node metastasis (yes/no) | 1.336 | 1.070-1.670 | 0.011 |  | 0.972 | 0.731-1.292 | 0.846 |
| Extrahepatic metastases (yes/no) | 1.258 | 0.996-1.588 | 0.054 |  |  |  |  |
| Abbreviations: HBV, hepatitis B virus; HCV, hepatitis C virus; ALBI, albumin-bilirubin; HSP90α, heat shock protein 90α; AFP, alpha fetoprotein; ALP, alkaline phosphatase; ALT, alanine aminotransferase; BCLC, Barcelona Clinic Liver Cancer; PVTT, portal vein tumor thrombus. | | | | | | | |

| **Supplementary Table 10. Univariate and multivariate Cox regression analysis of overall survival in surgical group** | | | | | | | |
| --- | --- | --- | --- | --- | --- | --- | --- |
|  | Univariable Cox regression | | |  | Multivariable Cox regression | | |
| Variable | HR | 95%CI | *p* |  | HR | 95%CI | *p* |
| Sex (male/female) | 1.132 | 0.63-2.034 | 0.679 |  |  |  |  |
| Age (≥65/<65 years) | 1.287 | 0.755-2.194 | 0.353 |  |  |  |  |
| HBV (positive/negative) | 0.890 | 0.534-1.484 | 0.655 |  |  |  |  |
| Alcoholism (positive/negative) | 1.194 | 0.710-2.008 | 0.503 |  |  |  |  |
| Diabetes mellitus (positive/negative) | 1.128 | 0.512-2.483 | 0.765 |  |  |  |  |
| Hypertension (positive/negative) | 1.877 | 1.067-3.302 | 0.029 |  | 1.658 | 0.910-3.019 | 0.098 |
| Child-Pugh class (B/A) | 2.397 | 1.348-4.261 | 0.003 |  | 1.886 | 0.971-3.662 | 0.061 |
| ALBI grade (2+3/1) | 1.396 | 0.808-2.413 | 0.232 |  |  |  |  |
| HSP90α (≥ 143.5/< 143.5 ng/ml) | 2.580 | 1.482-4.493 | 0.001 |  | 1.192 | 0.640-2.219 | 0.580 |
| AFP (≥400/<400 ng/ml) | 1.802 | 1.081-3.003 | 0.024 |  | 1.268 | 0.729-2.206 | 0.401 |
| ALP (≥125/<125 U/L) | 4.038 | 2.380-6.852 | < 0.001 |  | 2.966 | 1.690-5.205 | < 0.001 |
| Platelet (<100000/≥100000/μL) | 1.156 | 0.613-2.179 | 0.654 |  |  |  |  |
| ALT (≥40/<40U/L) | 1.436 | 0.858-2.405 | 0.168 |  |  |  |  |
| Leukocyte (<4000/≥4000/μL) | 2.207 | 0.882-5.518 | 0.091 |  |  |  |  |
| BCLC stage |  |  | < 0.001 |  |  |  | 0.099 |
| 0/A | 1.000 |  |  |  | 1.000 |  |  |
| B | 1.960 | 1.046-3.673 | 0.036 |  | 1.051 | 0.399-2.768 | 0.920 |
| C | 4.348 | 2.339-8.083 | < 0.001 |  | 2.010 | 0.857-4.714 | 0.108 |
| Number of tumor (≥2/<2) | 1.947 | 1.153-3.286 | 0.013 |  | 1.707 | 0.786-3.708 | 0.177 |
| Tumor diameter (≥5/<5 cm) | 1.811 | 1.072-3.060 | 0.026 |  | 1.544 | 0.821-2.901 | 0.177 |
| Abbreviations: HBV, hepatitis B virus; HCV, hepatitis C virus; ALBI, albumin-bilirubin; HSP90α, heat shock protein 90α; AFP, alpha fetoprotein; ALP, alkaline phosphatase; ALT, alanine aminotransferase; BCLC, Barcelona Clinic Liver Cancer; PVTT, portal vein tumor thrombus. | | | | | | | |

| **Supplementary Table 11. Univariate and multivariate Cox regression analysis of overall survival in TACE group** | | | | | | | |
| --- | --- | --- | --- | --- | --- | --- | --- |
|  | Univariable Cox regression | | |  | Multivariable Cox regression | | |
| Variable | HR | 95%CI | *p* |  | HR | 95%CI | *p* |
| Sex (male/female) | 1.156 | 0.880-1.520 | 0.297 |  |  |  |  |
| Age (≥65/<65 years) | 0.748 | 0.592-0.946 | 0.015 |  | 0.857 | 0.674-1.089 | 0.207 |
| HBV (positive/negative) | 1.114 | 0.916-1.355 | 0.280 |  |  |  |  |
| HCV (positive/negative) | 0.777 | 0.415-1.456 | 0.431 |  |  |  |  |
| Alcoholism (positive/negative) | 1.031 | 0.851-1.249 | 0.756 |  |  |  |  |
| Diabetes mellitus (positive/negative) | 1.089 | 0.783-1.514 | 0.612 |  |  |  |  |
| Hypertension (positive/negative) | 0.786 | 0.582-1.061 | 0.116 |  |  |  |  |
| Child-Pugh class (B/A) | 1.524 | 1.233-1.883 | < 0.001 |  | 1.279 | 1.008-1.622 | 0.043 |
| ALBI grade (2+3/1) | 1.318 | 1.060-1.640 | 0.013 |  | 1.040 | 0.816-1.325 | 0.754 |
| HSP90α (≥ 143.5/< 143.5 ng/ml) | 2.025 | 1.672-2.452 | < 0.001 |  | 1.630 | 1.319-2.013 | < 0.001 |
| AFP (≥400/<400 ng/ml) | 1.286 | 1.061-1.559 | 0.010 |  | 1.028 | 0.839-1.260 | 0.787 |
| ALP (≥125/<125 U/L) | 1.499 | 1.229-1.827 | < 0.001 |  | 1.143 | 0.922-1.418 | 0.222 |
| Platelet (<100000/≥100000/μL) | 0.953 | 0.775-1.172 | 0.648 |  |  |  |  |
| ALT (≥40/<40U/L) | 1.210 | 0.999-1.467 | 0.052 |  |  |  |  |
| Leukocyte (<4000/≥4000/μL) | 1.060 | 0.829-1.355 | 0.642 |  |  |  |  |
| BCLC stage |  |  | < 0.001 |  |  |  | 0.771 |
| 0/A | 1.000 |  |  |  | 1.000 |  |  |
| B | 1.403 | 0.975-2.020 | 0.068 |  | 0.963 | 0.612-1.516 | 0.872 |
| C | 2.115 | 1.523-2.936 | < 0.001 |  | 1.113 | 0.665-1.864 | 0.683 |
| Number of tumor (≥2/<2) | 1.696 | 1.288-2.233 | < 0.001 |  | 1.492 | 1.054-2.113 | 0.024 |
| Tumor diameter (≥5/<5 cm) | 1.156 | 0.933-1.433 | 0.186 |  |  |  |  |
| PVTT (positive/negative) | 1.606 | 1.325-1.946 | < 0.001 |  | 1.166 | 0.893-1.522 | 0.260 |
| Lymph node metastasis (yes/no) | 1.477 | 1.220-1.788 | < 0.001 |  | 1.040 | 0.790-1.370 | 0.778 |
| Extrahepatic metastases (yes/no) | 1.377 | 1.079-1.757 | 0.010 |  | 1.018 | 0.777-1.335 | 0.895 |
| Abbreviations: HBV, hepatitis B virus; HCV, hepatitis C virus; ALBI, albumin-bilirubin; HSP90α, heat shock protein 90α; AFP, alpha fetoprotein; ALP, alkaline phosphatase; ALT, alanine aminotransferase; BCLC, Barcelona Clinic Liver Cancer; PVTT, portal vein tumor thrombus; TACE, transcatheter arterial chemoembolization. | | | | | | | |

| **Supplementary Table 12. Univariate and multivariate Cox regression analysis of overall survival in adjuvant TACE group** | | | | | | | |
| --- | --- | --- | --- | --- | --- | --- | --- |
|  | Univariable Cox regression | | |  | Multivariable Cox regression | | |
| Variable | HR | 95%CI | *p* |  | HR | 95%CI | *p* |
| Sex (male/female) | 2.261 | 0.680-7.518 | 0.183 |  |  |  |  |
| Age (≥65/<65 years) | 1.075 | 0.474-2.440 | 0.862 |  |  |  |  |
| HBV (positive/negative) | 0.543 | 0.266-1.109 | 0.094 |  |  |  |  |
| Alcoholism (positive/negative) | 1.454 | 0.717-2.946 | 0.299 |  |  |  |  |
| Hypertension (positive/negative) | 0.298 | 0.090-0.987 | 0.048 |  | 0.348 | 0.104-1.171 | 0.088 |
| Child-Pugh class (B/A) | 1.029 | 0.310-3.416 | 0.963 |  |  |  |  |
| ALBI grade (2+3/1) | 1.411 | 0.693-2.872 | 0.343 |  |  |  |  |
| HSP90α (≥ 143.5/< 143.5 ng/ml) | 2.946 | 1.362-6.374 | 0.006 |  | 2.037 | 0.882-4.707 | 0.096 |
| AFP (≥400/<400 ng/ml) | 2.070 | 1.009-4.248 | 0.047 |  | 1.603 | 0.755-3.401 | 0.219 |
| ALP (≥125/<125 U/L) | 1.550 | 0.704-3.412 | 0.276 |  |  |  |  |
| Platelet (<100000/≥100000/μL) | 1.335 | 0.603-2.953 | 0.476 |  |  |  |  |
| ALT (≥40/<40U/L) | 1.250 | 0.614-2.547 | 0.538 |  |  |  |  |
| Leukocyte (<4000/≥4000/μL) | 3.193 | 0.751-13.573 | 0.116 |  |  |  |  |
| BCLC stage |  |  | 0.016 |  |  |  | 0.241 |
| 0/A | 1.000 |  |  |  | 1.000 |  |  |
| B | 0.906 | 0.405-2.029 | 0.810 |  | 1.029 | 0.456-2.319 | 0.946 |
| C | 3.351 | 1.289-8.710 | 0.013 |  | 2.316 | 0.823-6.513 | 0.111 |
| Number of tumor (≥2/<2) | 0.683 | 0.336-1.389 | 0.292 |  |  |  |  |
| Tumor diameter (≥5/<5 cm) | 1.812 | 0.871-3.772 | 0.112 |  |  |  |  |
| Abbreviations: HBV, hepatitis B virus; HCV, hepatitis C virus; ALBI, albumin-bilirubin; HSP90α, heat shock protein 90α; AFP, alpha fetoprotein; ALP, alkaline phosphatase; ALT, alanine aminotransferase; BCLC, Barcelona Clinic Liver Cancer; PVTT, portal vein tumor thrombus; TACE, transcatheter arterial chemoembolization. | | | | | | | |

| **Supplementary Table 13. Univariate and multivariate Cox regression analysis of overall  survival in ICI plus targeted therapy group in ICI plus targeted therapy group** | | | | | | | |
| --- | --- | --- | --- | --- | --- | --- | --- |
|  | Univariable Cox regression | | |  | Multivariable Cox regression | | |
| Variable | HR | 95%CI | *p* |  | HR | 95%CI | *p* |
| Sex (male/female) | 0.980 | 0.533-1.802 | 0.949 |  |  |  |  |
| Age (≥65/<65 years) | 0.947 | 0.489-1.836 | 0.872 |  |  |  |  |
| HBV (positive/negative) | 1.630 | 0.950-2.796 | 0.076 |  |  |  |  |
| Alcoholism (positive/negative) | 1.051 | 0.605-1.828 | 0.859 |  |  |  |  |
| Diabetes mellitus (positive/negative) | 0.756 | 0.342-1.674 | 0.491 |  |  |  |  |
| Hypertension (positive/negative) | 0.992 | 0.485-2.028 | 0.982 |  |  |  |  |
| Child-Pugh class (B/A) | 1.992 | 1.167-3.399 | 0.011 |  | 1.820 | 1.063-3.117 | 0.029 |
| ALBI grade (2+3/1) | 1.527 | 0.788-2.960 | 0.210 |  |  |  |  |
| HSP90α (≥ 143.5/< 143.5 ng/ml) | 2.473 | 1.449-4.223 | 0.001 |  | 1.002 | 1.000-1.005 | 0.047 |
| AFP (≥400/<400 ng/ml) | 2.085 | 1.219-3.568 | 0.007 |  | 1.427 | 0.788-2.583 | 0.240 |
| ALP (≥125/<125 U/L) | 1.571 | 0.867-2.845 | 0.136 |  |  |  |  |
| Platelet (<100000/≥100000/μL) | 1.063 | 0.586-1.927 | 0.841 |  |  |  |  |
| ALT (≥40/<40U/L) | 1.156 | 0.679-1.967 | 0.593 |  |  |  |  |
| Leukocyte (<4000/≥4000/μL) | 0.793 | 0.387-1.624 | 0.526 |  |  |  |  |
| BCLC stage |  |  | 0.058 |  |  |  |  |
| 0/A | 1.000 |  |  |  |  |  |  |
| B | 5.604 | 0.625-50.239 | 0.124 |  |  |  |  |
| C | 9.358 | 1.291-67.824 | 0.027 |  |  |  |  |
| Number of tumor (≥2/<2) | 1.964 | 0.888-4.346 | 0.096 |  |  |  |  |
| Tumor diameter (≥5/<5 cm) | 1.386 | 0.784-2.449 | 0.261 |  |  |  |  |
| PVTT (positive/negative) | 2.621 | 1.498-4.588 | 0.001 |  | 1.934 | 1.056-3.540 | 0.033 |
| Lymph node metastasis (yes/no) | 1.400 | 0.790-2.482 | 0.250 |  |  |  |  |
| Extrahepatic metastases (yes/no) | 1.592 | 0.936-2.708 | 0.086 |  |  |  |  |
| Abbreviations: HBV, hepatitis B virus; HCV, hepatitis C virus; ALBI, albumin-bilirubin; HSP90α, heat shock protein 90α; AFP, alpha fetoprotein; ALP, alkaline phosphatase; ALT, alanine aminotransferase; BCLC, Barcelona Clinic Liver Cancer; PVTT, portal vein tumor thrombus; ICI, immune checkpoint inhibitor. | | | | | | | |

| **Supplementary Table 14. Univariate and multivariate Cox regression analysis of overall survival in TACE plus ICI group** | | | | | | | |
| --- | --- | --- | --- | --- | --- | --- | --- |
|  | Univariable Cox regression | | |  | Multivariable Cox regression | | |
| Variable | HR | 95%CI | *p* |  | HR | 95%CI | *p* |
| Sex (male/female) | 0.619 | 0.303-1.262 | 0.187 |  |  |  |  |
| Age (≥65/<65 years) | 1.175 | 0.520-2.654 | 0.699 |  |  |  |  |
| HBV (positive/negative) | 0.765 | 0.388-1.508 | 0.440 |  |  |  |  |
| Alcoholism (positive/negative) | 0.825 | 0.446-1.523 | 0.538 |  |  |  |  |
| Diabetes mellitus (positive/negative) | 0.171 | 0.023-1.248 | 0.082 |  |  |  |  |
| Hypertension (positive/negative) | 0.790 | 0.309-2.021 | 0.623 |  |  |  |  |
| Child-Pugh class (B/A) | 1.583 | 0.830-3.019 | 0.163 |  |  |  |  |
| ALBI grade (2+3/1) | 1.097 | 0.548-2.196 | 0.794 |  |  |  |  |
| HSP90α (≥ 143.5/< 143.5 ng/ml) | 2.071 | 1.085-3.952 | 0.027 |  | 2.071 | 1.085-3.952 | 0.027 |
| AFP (≥400/<400 ng/ml) | 0.799 | 0.434-1.471 | 0.471 |  |  |  |  |
| ALP (≥125/<125 U/L) | 1.569 | 0.840-2.930 | 0.158 |  |  |  |  |
| Platelet (<100000/≥100000/μL) | 1.096 | 0.486-2.474 | 0.825 |  |  |  |  |
| ALT (≥40/<40U/L) | 0.890 | 0.484-1.636 | 0.708 |  |  |  |  |
| Leukocyte (<4000/≥4000/μL) | 0.555 | 0.244-1.262 | 0.160 |  |  |  |  |
| BCLC stage |  |  | 0.574 |  |  |  |  |
| 0/A | 1.000 |  |  |  |  |  |  |
| B | 0.866 | 0.206-3.644 | 0.844 |  |  |  |  |
| C | 0.607 | 0.186-1.987 | 0.410 |  |  |  |  |
| Number of tumor (≥2/<2) | 1.549 | 0.551-4.356 | 0.406 |  |  |  |  |
| Tumor diameter (≥5/<5 cm) | 1.834 | 0.709-4.744 | 0.211 |  |  |  |  |
| PVTT (positive/negative) | 0.843 | 0.458-1.550 | 0.582 |  |  |  |  |
| Lymph node metastasis (yes/no) | 1.054 | 0.556-1.998 | 0.872 |  |  |  |  |
| Extrahepatic metastases (yes/no) | 1.324 | 0.703-2.495 | 0.385 |  |  |  |  |
| Abbreviations: HBV, hepatitis B virus; HCV, hepatitis C virus; ALBI, albumin-bilirubin; HSP90α, heat shock protein 90α; AFP, alpha fetoprotein; ALP, alkaline phosphatase; ALT, alanine aminotransferase; BCLC, Barcelona Clinic Liver Cancer; PVTT, portal vein tumor thrombus; TACE, transcatheter arterial chemoembolization; ICI, immune checkpoint inhibitor. | | | | | | | |

| **Supplementary Table 15. Univariate and multivariate logistic regression analysis of HSP90α level** | | | | | | | |
| --- | --- | --- | --- | --- | --- | --- | --- |
|  | Univariable Cox regression | | |  | Multivariable Cox regression | | |
| Variable | OR | 95%CI | *p* |  | OR | 95%CI | *p* |
| Sex (male/female) | 1.423 | 1.131-1.790 | 0.003 |  | 0.983 | 0.732-1.319 | 0.909 |
| Age (≥65/<65 years) | 0.678 | 0.552-0.833 | < 0.001 |  | 0.767 | 0.594-0.991 | 0.042 |
| HBV (positive/negative) | 0.999 | 0.837-1.192 | 0.987 |  |  |  |  |
| HCV (positive/negative) | 0.789 | 0.417-1.493 | 0.467 |  |  |  |  |
| Alcoholism (positive/negative) | 1.219 | 1.020-1.457 | 0.029 |  | 1.148 | 0.914-1.443 | 0.236 |
| NAFLD (positive/negative) | 1.017 | 0.481-2.149 | 0.965 |  |  |  |  |
| Diabetes mellitus (positive/negative) | 0.717 | 0.524-0.980 | 0.037 |  | 0.938 | 0.638-1.378 | 0.743 |
| Hypertension (positive/negative) | 0.758 | 0.589-0.975 | 0.031 |  | 0.926 | 0.678-1.264 | 0.628 |
| Child-Pugh class (B+C/A) | 2.777 | 2.284-3.375 | < 0.001 |  | 1.605 | 1.242-2.073 | < 0.001 |
| ALBI grade (2+3/1) | 2.791 | 2.251-3.460 | < 0.001 |  | 1.604 | 1.227-2.096 | 0.001 |
| AFP (≥400/<400 ng/ml) | 2.987 | 2.489-3.583 | < 0.001 |  | 2.341 | 1.881-2.912 | < 0.001 |
| ALP (≥125/<125 U/L) | 4.555 | 3.741-5.546 | < 0.001 |  | 2.222 | 1.756-2.813 | < 0.001 |
| Platelet (<100000/≥100000/μL) | 1.834 | 1.487-2.263 | < 0.001 |  | 1.570 | 1.194-2.063 | 0.001 |
| ALT (≥40/<40U/L) | 2.242 | 1.871-2.688 | < 0.001 |  | 1.498 | 1.201-1.867 | < 0.001 |
| Leukocyte (<4000/≥4000/μL) | 2.412 | 1.840-3.160 | < 0.001 |  | 2.229 | 1.586-3.132 | < 0.001 |
| BCLC stage |  |  | < 0.001 |  |  |  | 0.379 |
| 0/A | 1.000 |  |  |  | 1.000 |  |  |
| B | 1.996 | 1.418-2.810 | < 0.001 |  | 0.926 | 0.577-1.485 | 0.750 |
| C | 7.475 | 5.579-10.014 | < 0.001 |  | 1.340 | 0.803-2.238 | 0.263 |
| D | 11.612 | 5.407-24.938 | < 0.001 |  | 1.487 | 0.594-3.723 | 0.396 |
| Number of tumor (≥2/<2) | 2.401 | 1.93-2.988 | < 0.001 |  | 1.320 | 0.948-1.837 | 0.100 |
| Tumor diameter (≥5/<5 cm) | 4.758 | 3.833-5.905 | < 0.001 |  | 2.688 | 2.081-3.472 | < 0.001 |
| PVTT (positive/negative) | 4.772 | 3.937-5.784 | < 0.001 |  | 1.944 | 1.451-2.603 | < 0.001 |
| Lymph node metastasis (yes/no) | 3.231 | 2.688-3.883 | < 0.001 |  | 1.189 | 0.883-1.602 | 0.255 |
| Extrahepatic metastases (yes/no) | 2.323 | 1.869-2.888 | < 0.001 |  | 1.098 | 0.823-1.467 | 0.524 |
| Abbreviations: HSP90α, heat shock protein 90α; HBV, hepatitis B virus; HCV, hepatitis C virus; NAFLD, nonalcoholic fatty liver disease; ALBI, albumin-bilirubin; AFP, alpha fetoprotein; ALP, alkaline phosphatase; ALT, alanine aminotransferase; BCLC, Barcelona Clinic Liver Cancer; PVTT, portal vein tumor thrombus. | | | | | | | |
